# Analysis of *Malassezia* Lipidome Disclosed Differences Among the Species and Reveals Presence of Unusual Yeast Lipids

**DOI:** 10.3389/fcimb.2020.00338

**Published:** 2020-07-15

**Authors:** Adriana Marcela Celis Ramírez, Adolfo Amézquita, Juliana Erika Cristina Cardona Jaramillo, Luisa F. Matiz-Cerón, Juan S. Andrade-Martínez, Sergio Triana, Maria Juliana Mantilla, Silvia Restrepo, Andrés Fernando González Barrios, Hans de Cock

**Affiliations:** ^1^Grupo de Investigación Celular y Molecular de Microorganismos Patógenos (CeMoP), Department of Biological Sciences, Universidad de los Andes, Bogotá, Colombia; ^2^Grupo de Ecofisiología, Comportamiento y Herpetología (GECOH), Department of Biological Sciences, Universidad de los Andes, Bogotá, Colombia; ^3^Grupo de Diseño de Productos y Procesos (GDPP), Chemical Engineering Department, Universidad de los Andes, Bogotá, Colombia; ^4^Research Group in Computational Biology and Microbial Ecology, Department of Biological Sciences, Universidad de los Andes, Bogotá, Colombia; ^5^Max Planck Tandem Group in Computational Biology, Universidad de los Andes, Bogotá, Colombia; ^6^Structural and Computational Biology Unit, European Molecular Biology Laboratory (EMBL), Heidelberg, Germany; ^7^Laboratorio de Micología y Fitopatología (LAMFU), Chemical Engineering Department, Universidad de los Andes, Bogotá, Colombia; ^8^Laboratorio de Micología y Fitopatología (LAMFU), Chemical and Food Engineering Department, Universidad de los Andes, Bogotá, Colombia; ^9^Grupo de Diseño de Productos y Procesos (GDPP), Chemical and Food Engineering Department, Universidad de los Andes, Bogotá, Colombia; ^10^Microbiology, Department of Biology, Faculty of Science, Institute of Biomembranes, Utrecht University, Utrecht, Netherlands

**Keywords:** lipidomic, ultra-high-pressure liquid chromatography/mass spectrometry, *Malassezia*, partial least squares discriminant analysis, fatty acids esters of hydroxyl fatty acids, diacylglyceryltrimethylhomoserine

## Abstract

*Malassezia* yeasts are lipid dependent and part of the human and animal skin microbiome. However, they are also associated with a variety of dermatological conditions and even cause systemic infections. How these yeasts can live as commensals on the skin and switch to a pathogenic stage has long been a matter of debate. Lipids are important cellular molecules, and understanding the lipid metabolism and composition of *Malassezia* species is crucial to comprehending their biology and host–microbe interaction. Here, we investigated the lipid composition of *Malassezia* strains grown to the stationary phase in a complex Dixon medium broth. In this study, we perform a lipidomic analysis of a subset of species; in addition, we conducted a gene prediction analysis for the detection of lipid metabolic proteins. We identified 18 lipid classes and 428 lipidic compounds. The most commonly found lipids were triglycerides (TAG), sterol (CH), diglycerides (DG), fatty acids (FAs), phosphatidylcholine (PC), phosphatidylethanolamine (PE), ceramides, cholesteryl ester (CE), sphingomyelin (SM), acylcarnitine, and lysophospholipids. Particularly, we found a low content of CEs in *Malassezia furfur*, atypical *M. furfur*, and *Malassezia pachydermatis* and undetectable traces of these components in *Malassezia globosa, Malassezia restricta*, and *Malassezia sympodialis*. Remarkably, uncommon lipids in yeast, like diacylglyceryltrimethylhomoserine and FA esters of hydroxyl FAs, were found in a variable concentration in these *Malassezia* species. The latter are bioactive lipids recently reported to have antidiabetic and anti-inflammatory properties. The results obtained can be used to discriminate different *Malassezia* species and offer a new overview of the lipid composition of these yeasts. We could confirm the presence and the absence of certain lipid-biosynthesis genes in specific species. Further analyses are necessary to continue disclosing the complex lipidome of *Malassezia* species and the impact of the lipid metabolism in connection with the host interaction.

## Introduction

Lipid-dependent *Malassezia* species belong to the phylum Basidiomycota and are the most important constituent of the human skin mycobiota. *Malassezia* species have been associated with dermatological conditions, such as dandruff/seborrheic dermatitis, pityriasis versicolor, and atopic dermatitis, and with more severe conditions, like systemic infections and pancreatic cancer (Grice and Dawson, 2017; Theelen et al., 2018; Aykut et al., 2019).

The absence of *de novo* synthesis of fatty acids (FAs) in *Malassezia* species is determined by the absence of genes that encode for FA synthase in their genomes (Triana et al., 2015; Wu et al., 2015; Lorch et al., 2018). This characteristic is related to the requirement to exploit lipid sources contained in the human sebum [triglycerides (TAG), FAs, wax esters, sterol esters, cholesterol, cholesterol esters, and squalene] (Ro and Dawson, 2005). For this reason, *Malassezia* species secrete several enzymes, such as esterases, lipases, lipoxygenases, and proteases, in order to supply their lipid requirements (Mayser and Gaitanis, 2010; Park et al., 2017).

This yeast can metabolize or modify FAs to carry out a variety of important biological processes, such as membrane biogenesis, energy homeostasis, energy storage, and metabolism, to carry out signal transduction and to contribute to fungal pathogenicity (Ro and Dawson, 2005; Celis Ramirez et al., 2017). Thus, changes in the external FA composition represent a challenge for *Malassezia* metabolism; however, not much is known about the lipid composition and adaptation of species of this genus (Shifrine and Marr, 1963; Porro et al., 1976; Huang et al., 1993; Mayser et al., 1998).

Considering the relevance and the complexity of lipid metabolism and potential applications of lipids, the emerging field into the omics known as lipidomics (as a subset of metabolomics) has been developed, and its application (Ibáñez et al., 2017), sensitivity, and reliability have increased in recent years along with the rapid advancement in mass spectrometry (MS) techniques (Wenk, 2005; Han, 2016; Yang and Han, 2016). The lipidome of the yeast *Saccharomyces cerevisiae* is well-characterized, and it has been widely used as an experimental system for studying lipid-related processes (Ejsing et al., 2009; Klose et al., 2012). Furthermore, lipidomic analyses have been carried out in various fungal pathogens, such as *Cryptococcus* species, *Candida* species, and *Paracoccidioides brasiliensis*, to investigate aspects related to virulence, antifungal resistance, and new antifungal targets (Hein and Hayen, 2012; Longo et al., 2013; Singh et al., 2017; Zamith-Miranda et al., 2019). Factors that affect lipid composition have also been studied in oleoginoseous yeasts in order to efficiently obtain biofuels (Beopoulos et al., 2011; Pomraning et al., 2015). Recently, differences in lipid profiles were detected via Raman spectroscopy and used to differentiate three *Malassezia* species (Petrokilidou et al., 2019); nonetheless, to date, no lipid profiles of *Malassezia* species based on MS have been reported.

The present study implemented lipidomics in combination with an *in silico* genomic analysis to investigate the lipid composition and synthesis of *Malassezia furfur*, atypical *M. furfur, Malassezia pachydermatis, Malassezia globosa, Malassezia restricta*, and *Malassezia sympodialis* after growth in complex Dixon medium broth.

## Materials and Methods

### Strains and Culture Conditions

The reference *Malassezia* strains—*M. furfur* CBS 1878, *M. globosa* CBS 7986, *M. pachydermatis* CBS 1879, *M. restricta* CBS 7877, and *M. sympodialis* CBS 7222 (Westerdijk Institute, Utrecht, The Netherlands)—and a previously reported isolate of *M. furfur* with atypical assimilation of Tween 80 (hereafter referred to as atypical *M. furfur*) (González et al., 2009) were precultured at 33°C using modified Dixon agar [mDixon agar; 36 g L^−1^ mycosel agar [BD, Franklin Lakes, NJ, USA], 20 g L^−1^ Ox bile, 36 g L^−1^ malt extract [Oxoid, Basingstoke, UK], 2 ml L^−1^ glycerol, 2 ml L^−1^ oleic acid, and 10 ml L^−1^ Tween 40] (Guého-Kellermann et al., 2010).

After 5 days of growth on mDixon agar, the yeast cells were suspended in 3 ml of distilled water with 0.1% Tween 80 used to inoculate 27-ml mDixon broth [20 g L^−1^ Ox bile, 6 g L^−1^ peptone [BD], 36 g L^−1^ malt extract [Oxoid, Basingstoke, UK], 2 ml L^−1^ glycerol, 2 ml L^−1^ oleic acid, and 10 ml L^−1^ Tween 40 and 500 mg L^−1^ chloramphenicol] that was grown at 33°C with 180 rpm to reach the stationary phase (Guého-Kellermann et al., 2010).

### Lipidomic Analysis

Aliquots of *Malassezia* species in the stationary phase were washed three times with phosphate-buffered saline with intermediate centrifugation at 1,248 g for 10 min. Cells were resuspended in 10 ml of phosphate-buffered saline and disrupted with a sonicator (Sonic Vibra Cell, Newtown, CT, USA) at 40% amplitude, performing the following procedure 10 times: 1 min sonication and 30 s of cooling on ice. The disrupted cells were centrifuged at 4°C at 5,000 g for 10 min, and the pellet was washed with deionized water and centrifuged using the same conditions. Each sample was freeze-dried and weighted before analysis.

The lipidomic analysis was performed at the West Coast Metabolomic Center (University of California at Davis). Lipid extraction was conducted according to the center's workflow involving sample extraction based on the “Matyash” method (Matyash et al., 2008) with some modifications, as described. Extraction was carried out using a biphasic solvent system of cold methanol, methyl-tert-butyl ether (MTBE), and water. The extraction was followed by ultra-high-pressure liquid chromatography (UHPLC), and the chromatographic analysis was performed as reported by Cardona Jaramillo et al. (2019). The separation was carried out in a Waters charged surface hybrid (CSH™) column, UHPLC CHS C18 (100 mm × 2.1 mm × 1.7 μm; Waters Corporation, MA, USA). The lipid detection was achieved with an Agilent 6530 quadrupole time-of-flight (QTOF) mass spectrometer with resolution *R* = 10,000 for positively charged lipids and with an Agilent 6550 QTOF mass spectrometer with resolution *R* = 20,000 for negatively charged lipids. The mobile phase A was a 90:10 mixture of isopropyl alcohol and acetonitrile with 10-mM ammonium formate and 0.1% formic acid. The mobile phase B was a 60:40 mixture of acetonitrile and water with 10 mM of ammonium formate and 0.1% formic acid. The elution gradient was 0 min 15% (A), 0–2 min 30% (A), 2–2.5 min 48% (A), 2.5–11 min 82% (A), 11–11.5 min 99% (A), 11.5–12 min 99% (A), 12–12.1 min 15% (A), and 12.1–15 min 15% (A). Raw data were processed qualitatively by Agilent's MassHunter software. Peak alignment was performed using MassProfiler Professional. MS/MS information and the LipidBlast library were used to identify the lipid compounds. A unique ID was given to each lipid, based on its retention time and mass–charge ratio, and an additional manual verification was made. Lipids were identified based on MS/MS fragmentation patterns using in-house Lipidblast software (Kind et al., 2013). Lipid peak heights were normalized using the yeast biomass weight, and the concentration of each metabolite in terms of %mol was calculated using the internal standard method when possible [ceramide C17 for ceramides; CE [22:1] for CE; DG [12:0/12:0/0:0] for DG; LPC [17:0] for lysophosphatidyl esters; MG [17.0/0:0/0:0] for monoglycerides; PC [25:0] for PC; PE [17:0/17:0] for PE; TAG [17:0/17:1/17:0] for TAG] (Supplementary Table 1). Lipidomic analyses resulting from six biological replicates were analyzed.

### Statistical Analysis

To test whether *Malassezia* strains can be discriminated based on (a) the whole chromatographic profiles and (b) the sole basis of the FAHFA lipids, we built partial least squares models coupled with discriminant analyses (PLS-DA). Briefly, the relative amount of lipid compounds was used as a predictor of a categorical variable, the strain. The use of DA alone would lead to spurious predictive models for two main reasons. First, groups of lipid compounds tend to co-occur throughout the analyzed samples, which leads to significant correlation between them and, thereby, to multicollinearity, an undesirable property for a set of predictors. Second, the number of predictors was very large (above 400 compounds for the whole lipidome and 77 for the FAHFA lipids) and far larger than the number of analyzed samples (36, six strains each repeated six times). The PLS model reduces the number of predictors to a lower number of uncorrelated (i.e., orthogonal) variables, which are then used to discriminate (DA) among categories (strains). PLS is related to principal component analysis (PCA). Whereas, PCA reduces the dimensionality of predictors based on the sole covariation between them, PLS further considers the ability of the new components to predict the output variables.

To estimate the lowest number of components that suffice to discriminate among strains, we first fitted complete PLS-DA models and then assessed the classification performance of our models. We used unscaled maximal multivariate distances because all descriptors (compound concentrations) were expressed in the same units. The performance curve was used to visualize the contribution of each additional principal component (PC) to reduce the error rate of classifications to the point that adding new components represented negligible (i.e., statistically non-significant) contributions. As expected, both overall error rate and balanced error rate in classification decreased with the number of components and then stabilized after the fifth (all lipids) or eighth (FAHFA lipids) PC. Therefore, we used five and eight PCs, respectively, in the following analyses.

Because PLS-DA deals with a high ratio of variables to samples, it may eventually lead to good classifications by chance (Gromski et al., 2015). To reduce the risk of overfitting (tendency to overfit), we refrained from reporting both the PLS-DA score plots obtained from the training data (Szymańska et al., 2011; Brereton and Lloyd, 2014; Gromski et al., 2015) and the R^2^, Q^2^, and DQ^2^ statistics (Szymańska et al., 2011; Gromski et al., 2015). Instead, we report here the number of misclassified cases (NMC) and the area under the receiver operating characteristic (AUROC) (Szymańska et al., 2011). Finally, we fitted two sparse versions of the model (sPLS-DA), which were intended to identify the subset of uncorrelated lipid compounds that best discriminated the strains and to eliminate the uninformative lipids. The number and identity of the lipids represented by each component was selected by cross-validation with 5-folds (groups) and 200 repetitions each, which rendered 1,000 permutations. M-fold values between 5 and 10 have been empirically shown to estimate relatively unbiased and stable error rates (Rohart et al., 2017). We then plotted the decrease in classification performance (error rate) with regard to the number of selected descriptors for each PC and checked both the load of compounds to the PCs as well as the stability of PC classification performance after adding compounds. All analyses were conducted on the package mixOmics (Rohart et al., 2017), as implemented in R^1^ (details of the code are given in S4 File).

### Gene Prediction and Annotation

Among the species considered, only those of *M. restricta* and *M. pachydermatis* had their associated proteome sequences uploaded to RefSeq as of June 2019, with 3,742 and 2,960 sequences, respectively (Park et al., 2017; Triana et al., 2017). These sequences were downloaded from the National Center for Biotechnology Information (NCBI) in June 2019, using their respective protein name with the basic query “(((scientific name) NOT partial) NOT hypothetical),” where “scientific name” took the values *Malassezia restricta* or *Malassezia pachydermatis*. For *M. sympodialis, M. globosa, M. furfur*, and atypical *M. furfur*, the previously annotated and experimentally validated proteins from Xu et al. (2007), Gioti et al. (2013) and Triana et al. (2017) were used.

For detection of lipid metabolism proteins, associated sequences for each lipid metabolism gene were downloaded from NCBI in June 2019. The search was conducted in three stages: first, only using RefSeq entries and hits from Basidiomycetes (taxID:5204). If this stage yielded less than four sequences, the possible hits were expanded to those coming from all fungi (taxID:4751). If this approach yielded <10 sequences, then all NCBI databases were considered, and duplicates were removed manually.

A custom BLAST protein database with the downloaded sequences was created using BLAST V.2.6.0+ (Camacho et al., 2009). For hidden Markov model (HMM), sequences associated with a given protein were aligned with Muscle V3.81 (Edgar, 2004) and then used to create an HMM with HMMER V.3.1 (Eddy, 1998). Lipid metabolism proteins and their respective ECs are presented in Table 1.

**Table 1:** Predicted *Malassezia* homologs of enzymes involved in lipid biosynthesis.

**Gene**	**EC number**	**Function**	**MF**	**AMF**	**MP**	**MS**	**MG**	**MR**
**Lipid synthesis**								
*FAS1*	EC 3.1.2.14	Fatty acid synthase (β subunit)	**X**	**X**	**X**	**X**	**X**	**X**
*FAA1, FAA2, FAA3, FAA4, FAT1*	EC 6.2.1.3	Long-chain fatty acyl-CoA synthetase						
*GPT2 (GAT1)*	EC 2.3.1.15	Glycerol-3-phosphate/dihydroxyacetone phosphate sn-1 acyltransferase						
*SCT1 (GAT2)*	EC 2.3.1.42	Glycerol-3-phosphate/dihydroxyacetone phosphate sn-1 acyltransferase						
*AYR1*	EC 1.1.1.101	1-Acyl-DHAP reductase						
*ALE1/SLC1/SLC4*	EC 2.3.1.51/EC 2.3.1.23	1-acyl G-3-P acyltransferase (lyso-phospholipid acyltransferase)						
*CDS1*	EC 2.7.7.41	Phosphatidate cytidylyltransferase						
*PAH1*	EC 3.1.3.4	Phosphatidate phosphatase						
*CHO1*	EC 2.7.8.8	CDP-diacylglycerol–serine O-phosphatidyltransferase						
*PSD1*	EC 4.1.1.65	Phosphatidylserine decarboxylase						
*PSD2*	EC 4.1.1.65	Phosphatidylserine decarboxylase						
*CHO2*	EC 2.1.1.17	Phosphatidylethanolamine N-methyltransferase				**X**	**X**	
*OPI3*	EC 2.1.1.71	Phosphatidyl-N-methylethanolamine N-methyltransferase				**X**		
*EKI1*	EC 2.7.1.82	Ethanolamine kinase	**X**	**X**	**X**	**X**	**X**	**X**
*CKI1*	EC 2.7.1.32	Choline kinase	**X**	**X**		**X**	**X**	**X**
*PCT1*	EC 2.7.7.15	Cholinephosphate cytidylyltransferase						
*CPT1*	EC 2.7.8.2	Cholinephosphotransferase						
*VPT29*	EC 2.7.1.137	Phosphatidylinositol 3-kinase						
*PIS1*	EC 2.7.8.11	Phosphatidylinositol synthase				**X**		
*LSB6*	EC 2.7.1.67	1-phosphatidylinositol 4-kinase						
*PIK1*	EC 2.7.1.67	1-phosphatidylinositol 4-kinase						
*STT4*	EC 2.7.1.67	1-phosphatidylinositol 4-kinase						
*MSS4*	EC 2.7.1.68	1-phosphatidylinositol-4-phosphate 5-kinase						
*FAB1*	EC 2.7.1.150	1-phosphatidylinositol-3-phosphate 5-kinase						
*PGS1*	EC 2.7.8.5	Phosphatidylglycerolphosphate synthase						
*GEP4*	EC 3.1.3.27	Phosphatidylglycerophosphatase			**X**			
*CRD1*	EC 2.7.8.41	Cardiolipin synthase						
*PLB1*	EC 3.1.1.5	PC/PE specific phospholipase B						
*SPO14*	EC 3.1.4.4	Phospholipase D						
*PLC1*	EC 3.1.4.11	Phospholipase C				**X**		
*DGK1*	EC 2.7.1.107	Diacylglycerol kinase	**X**	**X**	**X**	**X**	**X**	**X**
*DGA1*	EC 2.3.1.15 EC 2.3.1.42	Diacylglycerol acyltransferase						
*LRO1*	EC 2.3.1.158	Phospholipid:DAG acyltransferase				**X**		
*ARE1*	EC 2.3.1.26	Acyl-CoA:cholesterol acyltransferase					**X**	
*ARE2*	EC 2.3.1.26	Acyl-CoA:cholesterol acyltransferase					**X**	
*ELO1, ELO2, ELO3*	EC 2.3.1.199	Elongases						
*IIFA38*	EC 1.1.1.330	β-keto acyl-CoA reductase/very-long-chain 3-oxoacyl-CoA reductase						
*PHS1*	EC 4.2.1.134	3-Hydroxy acyl-CoA dehydratase						
*TSC3*	EC 1.3.1.93	Enoyl-CoA reductase						
*INO1*	EC 5.5.1.4	Inositol 3-P synthase						
*INM1*	EC 3.1.3.25	Inositol-phosphate phosphatase						
*OLE1*	EC 1.14.19.2	Δ9-desaturase					**X**	**X**
*ECI1*	EC 5.3.3.8	Δ^3, 2^-enoyl-CoA isomerase	**X**	**X**	**X**	**X**	**X**	**X**
*SPS19*	EC 1.3.1.34	2,4-dienoyl-CoA reductase						
*DCI1*	EC 5.3.3	Delta(3,5)-delta(2,4)-dienoyl-CoA isomerase						
*FAD2/3*	EC 1.3.1.35	Δ12-desaturase/ω3-desaturase						
*YJU3*	EC 3.1.1.23	Acylglycerol lipase						
*TGL3*	EC 3.1.1.3	Bifunctional triacylglycerol lipase and LPE acyltransferase						
*TGL4*	EC 3.1.1.3	Multifunctional lipase/hydrolase/phospholipase						
*TGL5*	EC 3.1.1.3	Bifunctional triacylglycerol lipase and LPA acyltransferase						
*TGL1*	EC 3.1.1.13	Steryl ester hydrolase						
*YEH1*	EC 3.1.1.13	Steryl ester hydrolase						
*YEH2*	EC 3.1.1.13	Steryl ester hydrolase						
*DPP1*	EC 3.1.3.81	Diacylglycerol pyrophosphate phosphatase 1			**X**	**X**		
*APP1*	EC 3.1.3.4	Phosphatidate phosphatase		**X**	**X**	**X**	**X**	**X**
*LPP1*	EC 3.1.1.-	Lipid phosphate phosphatase 1		**X**	**X**	**X**	**X**	**X**
**Glycosphingolipid synthesis**								
*LCB1/2*	EC 2.3.1.50	Serine palmitoyltransferase						
*TSC10*	EC 1.1.1.102	3-ketosphinganine reductase						
*LAC1/LAG1*	EC 2.3.1.24	Sphingosine N-acyltransferase						
*YDC1*	EC 3.5.1	Alkaline dihydroceramidase						
*SUR2*	EC 1	Sphinganine C4-hydroxylase						
*YPC1*	EC 3.5.1	Alkaline ceramidase						
*SCS7*	EC 1.14.18.-	Sphingolipid alpha-hydroxylase						
*LCB4*	EC 2.7.1.91	Sphingoid long-chain base kinase						
*LCB3*	EC 3.1.3	Long-chain base-1-phosphate phosphatase						
*YSR3*	EC 3.1.3	Dihydrosphingosine 1-phosphate phosphatase			**X**	**X**	**X**	**X**
*DPL1*	EC 4.1.2.27	Dihydrosphingosine phosphate lyase						
*AUR1*	EC 2	Phosphatidylinositol:ceramide phosphoinositol transferase						
*CSH1/CSG1*	EC 2.4	Mannosylinositol phosphorylceramide (MIPC) synthase catalytic subunit						
*IPT1*	EC 2	Inositolphosphotransferase						
**Sterol synthesis enzymes**								
*ERG10*	EC 2.3.1.9	Acetyl-CoA C-acetyltransferase						
*ERG13*	EC 2.3.3.10	3-hydroxy-3-methylglutaryl-CoA (HMG-CoA) synthase						
HMG1/2	EC 1.1.1.34	HMG-CoA reductase						
ERG12	EC 2.7.1.36	Mevalonate kinase						
ERG8	EC 2.7.4.2	Phosphomevalonate kinase						
ERG19	EC 4.1.1.33	Mevalonate pyrophosphate decarboxylase						
IDI1	EC 5.3.3.2	Isopentenyl diphosphate:dimethylallyl diphosphate isomerase						
ERG20	EC 2.5.1.10	Farnesyl pyrophosphate synthetase						
ERG9	EC 2.5.1.21	Farnesyl-diphosphate farnesyl transferase (squalene synthase)						
ERG1	EC 1.14.13.132	Squalene epoxidase						
ERG7	EC 5.4.99.7	Lanosterol synthase						
ERG11	EC 1.14.13.70	Sterol 14 α-demethylases						
ERG24	EC 1.3.1.70	Sterol C-14 reductases						
ERG25	EC 1.14.13.72	Sterol C-4 methyl oxidases						
ERG26	EC 1.1.1.170	Sterol C-4 decarboxylases			**X**	**X**	**X**	**X**
ERG27	EC 1.1.1.270	Sterol C-3 dehydrogenase				**X**		
ERG6	EC 2.1.1.41	Sterol C-24 methyltransferases						
ERG2	EC 5.3.3.5	Sterol C-8 isomerases				**X**		
ERG3	EC 1.14.19.20	Sterol C-5 desaturases				**X**		
ERG4	EC 1.3.1.71	Sterol C-24 reductases						
ERG5	EC 1.14.19.41	Sterol C-22 desaturases						
DHCR7	EC1.3.1.21	7-dehydrocholesterol reductase						
**Acetylation/deacetylation (Sterol acetates formation)**								
*ATF2*	EC 2.3.1.84	Alcohol O-acetyltransferase 1	**X**	**X**	**X**	**X**	**X**	**X**
*SAY1*	EC 3.1.1	Steryl acetyl hydrolase 1						

*Corresponding gene in: MF, Malassezia furfur CBS1878; AMF, atypical Malassezia furfur; MP, Malassezia pachydermatis CBS1879; MS, Malassezia sympodialis CBS7222; MG, Malassezia globosa CBS7986; MR, Malassezia restricta CBS7877; EC number, enzyme commission number. X indicate absence*.

The protein sequences of the six *Malassezia* species were subjected to three analyses. First, they were blasted against the aforementioned BLAST database. Next, they were scanned for HMMs against the HMM database. Finally, both the *Malassezia* sequences and all downloaded, annotated proteins retrieved from NCBI were subjected to an orthologous group analysis using OrthoFinder V.2.3.3 (Emms and Kelly, 2015). In that context, a *Malassezia* proteome was suspected to harbor one of the lipid metabolism proteins if it fulfilled one of three conditions: (1) It had a BLAST hit against one of the sequences associated with the corresponding protein, with an average nucleotide identity score of 60% or higher and an e-value of 1e-5 or less. (2) It was grouped by OrthoFinder in an orthologous cluster associated with the same protein. Here, an orthologous cluster was considered to be associated with a given protein if at least 60% of the downloaded sequences from that protein were present and if they constituted at least 40% of the total number of sequences in the cluster. Note that the orthologous clusters were created *de novo* with OrthoFinder using the lipid metabolism and *Malassezia* sequences. (3) It had a HMM that can hit against the HMM associated with that protein, with a coverage of at least 60% of the protein and an e-value of 1e-5 or less. All *Malassezia* sequences that fulfilled these criteria were subjected to a final verification in which their domains and motifs were identified with InterProScan (Jones et al., 2014) and compared with those harbored by the proteins that were downloaded from NCBI. Note that the conditions in question could be fulfilled by separate proteins from the same genome, in which case both were further analyzed with InterPro.

InterProScan verification was the definitive criterion for asserting that a given lipid metabolism protein was present in a genome. Given the variations in the length of some proteins from *Malassezia* species as compared with the downloaded and annotated lipid metabolism genes, in some cases, the HMM criterion was relaxed to 40% coverage if no criteria were fulfilled by any sequence in a given species with the original thresholds. Any hits obtained with the new 40% coverage criterion were then verified with InterPro as described previously.

## Results

### Lipid Profiles of *Malassezia* Strains

**Figure 1:**
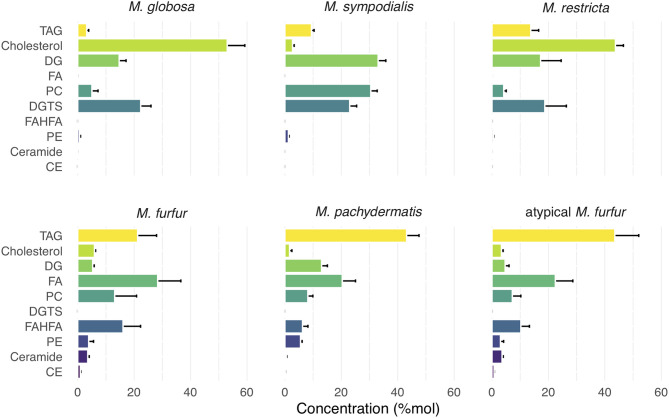
Relative concentration of major lipid classes in five species of *Malassezia* and a putative *M. furfur* (atypical *M. furfur*) strain. Lipid classes are organized top-down according to the average concentration throughout all species. Species are organized from left to right according to the decreasing concentration of triacylglycerols (TAG) lipids. Each lipid class is expressed in %mol by their relative molar contribution to total lipids. Cholesterol should be read as: cholesterol and closely related sterols that could currently not be resolved by the technique used. Bars and lines denote mean and standard deviation (SD), respectively. TAG, triglycerides; DG, diglycerides; FA, fatty acids; PC, phosphatidylcholine; DGTS, diacylgyceryltrimethylhomoserine; FAHFA, fatty acids hydroxyl fatty acids; PE, phosphatidylethanolamine; CE, cholesteryl ester.

Using ultra-high-performance liquid chromatography combined with quadrupole time-of-flight mass spectrometry (UHPLC/QTOF-MS) (Köfeler et al., 2012), we identified 18 lipid classes and 428 lipidic compounds. Lipid identification was performed by combining the retention time, the exact precursor mass, and the product ion spectra. The lipid classes identified are as follows: TAG, sterols (indicated in Figure 1 as cholesterol), diglycerides (DG), FAs, phosphatidylcholine (PC), diacylglyceryltrimethylhomoserine (DGTS), fatty acids esters of hydroxyl fatty acids (FAHFAs), phosphatidylethanolamine (PE), ceramides, cholesteryl ester (CE), and ceramide or sphingomyelin (SM); the relative abundance (%mol) of these species differed between the six species analyzed (Figure 1). Remarkably, we observed an unusually high relative amount of the neutral lipids' sterols and TAG, and *M. furfur*, atypical *M. furfur*, and *M. pachydermatis* that contained more TAG and *M. globosa, M. sympodialis*, and *M. restricta* that contained more sterols. The latter three contained more sterol as compared with TAG. We detected sterols, but we could not distinguish among the different sterol species in fungi based on the currently used analysis (Weete et al., 2010). TAG was the most abundant among *M. furfur*, atypical *M. furfur*, and *M. pachydermatis*, followed by FA, FAHFAs, PE, ceramides, and CE. In contrast, *M. globosa, M. restricta*, and *M. sympodialis* were characterized by the presence of sterols, DG, PC, and DGTS. A low concentration of CE was found in *M. furfur*, atypical *M. furfur*, and *M. pachydermatis*, and these were undetectable in the lipidome of *M. globosa, M. restricta*, and *M. sympodialis*. In *M. sympodialis*, a low concentration of sterols was detected similarly in *M. furfur*, atypical *M. furfur*, and *M. pachydermatis* (Figure 1). Phosphatidylinositol (PI) were not identified in the current analysis.

The FAs detected had chain lengths of 11–28, including even-chain and odd-chain FAs. Odd chain FAs found would be the α-oxidation products (Řezanka and Sigler, 2009). Precursors of eicosanoid FAs, such as longer PUFAs, including arachidonic (20:4) and docosahexaenoic acid DHA (22:6), were also detected (Supplementary Table 1). Glycerophospholipids were characterized by the presence of PA, PC, PE, and PG (Figure 1; Supplementary Table 2). Particularly, we found a higher concentration of PC in *M. sympodialis, M. furfur, M. pachydermatis*, and atypical *M. furfur* in comparison with that in *M. globosa* and *M. restricta*. *M. pachydermatis, M. furfur*, and atypical *M. furfur* had higher PE levels compared with *M. sympodialis, M. globosa*, and *M. restricta*. Besides PS, phosphatidylglycerol (PG) and cardiolipin were also detected but in a low concentration (data not shown). We also detected lysophosphatidylcholine, lysophosphatidylethanolamine, and lysophosphatidylinositol (Supplementary Table 2).

A subset of the FAs detected were those that are linked via a hydroxyl group to a second FA (FAHFA), of which we detected 77 different molecular species (Supplementary Table 2). Our analysis showed the presence of a wide variety of FAHFA in variable concentrations in *Malassezia* strains (Figure 1; Supplementary Table 3). In *M. furfur*, atypical *M. furfur*, and *M. pachydermatis*, FAHFA species were found in higher amounts. In contrast, for *M. globosa, M. restricta*, and *M. sympodialis*, approximately a 100-fold lower amount was detected when compared with the other three *Malassezia* species (Supplementary Table 1).

FA moieties of FAHFAs were mainly represented by palmitic acid, palmitoleic acid, stearic acid, oleic acid, linoleic acid, linolenic acid, and arachidonic acid, and less frequently by saturated FAs like capric acid, undecylic acid, mysristic acid, lauric acid, and PUFAs such as dihomo-γ-linolenic acid and docosahexaenoic acid (DHA) (Supplementary Table 2). We could not determine the fragments corresponding to the hydroxylation position of hydroxy FA because this cannot be determined precisely via the MS/MS data obtained (Zhu et al., 2018). Based on previous investigations by Wilde and Steward, however, a hydroxyl group at the 9th position can be expected since 9-hydroxypalmitic acid was shown to be the major product of metabolism of *Pityrosporum ovale* and 9-hydroxystearic to a lower extent (Wilde and Stewart, 1968). Although low amounts of DGTS (34:1), (34:2), (34:3), and (36:3) were detected, these compounds were characteristically present in *M. globosa, M. restricta*, and *M. sympodialis* and absent in *M. furfur*, atypical *M. furfur*, and *M. pachydermatis*.

We did not perform a lipidomic analysis of mDixon broth only to address the possibility that some of the lipids in the medium associated to cells. However, the lipidome profiles showed clear differences among the species analyzed, indicating that similar amounts of lipids are not simply carried over to all strains. Further analysis is necessary to rule out specific association of certain lipid species in this media to the *Malassezia* species.

### Discrimination of *Malassezia* Strains

**Figure 2:**
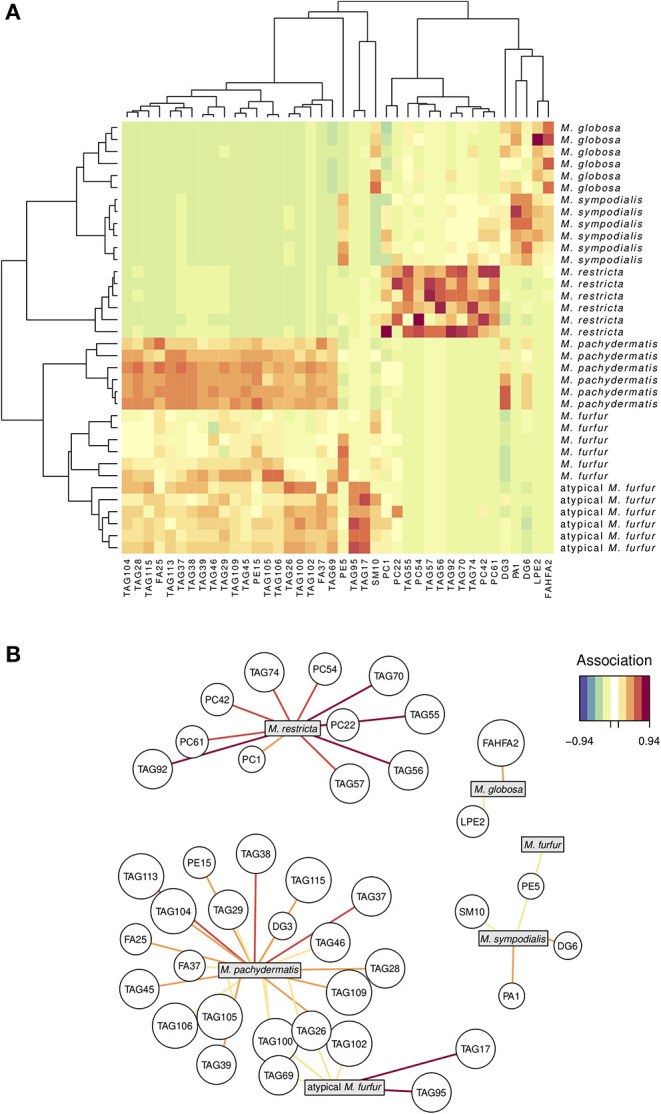
Lipidomic signature in five species of *Malassezia* and a putative *M. furfur* (atypical *M. furfur*) strain, six biological replicates were analyzed. A sparse least partial square analysis coupled to a discriminant analysis (sPLS-DA) reduced the dimensionality of 428 to 40 compounds, which successfully discriminated among the studied lineages. Above **(A)**, both species and compound clustering according to the similarity in lipid profiles; the heat map denotes higher (red) to lower (green-blue) concentrations of each compound. Below **(B)**, network visualization of the correlation between each species and the main compounds that characterize it; both positive and negative correlations with magnitude below 0.52 are not included for clarity. The color of the line denotes the magnitude of the correlation coefficient.

We next investigated whether we could discriminate the six *Malassezia* species examined in this study using the lipid profiles in which we identified 428 molecular species (Supplementary Tables 1, 2). For example, DG1 represents the molecular species of diacylglycerol, with two FA species represented as 32:0. Using the 428 molecular species as the lipid profiles, we performed an sPLS-DA and reduced the dimensionality of 428 original molecular species to 40 relevant compounds, represented by five orthogonal PCs (Figure 2). These compounds successfully discriminated the six *Malassezia* strains used in this study, with an error rate of 0.017 and no misclassified cases (NMC = 0) (Figure 2A). Hence, the AUROC was 1.0 (*p* = 0.0013) when classifying each species against all others. The hierarchical clustering resulting from the sPLS-DA (Figure 2A) showed two evident species clusters based on lipid profiles. The first joined *M. globosa* with *M. sympodialis* and then both to *M. restricta*. The second included *M. pachydermatis* and the two strains of *M. furfur* (Figure 2A). The clustering approach distinguished particularly well among species based on lipids, like TAG, cholesterol, FA, PC, DGTS, FAHFAs, PE, ceramides, and CE (Figure 2A).

We used the molecular species data set (Supplementary Table 1) and the network graphic representation of the sPLS-DA (Figure 2B) to perform a more detailed exploration of which molecular species appear associated with certain *Malassezia* species. Four out of the six *Malassezia* species revealed positive associations with a unique compound. The other two were both associated with four TAG (69, 100, 26, and 102) compounds, but the atypical *M. furfur* was strongly and uniquely associated with TAG17 and TAG95, whereas *M. pachydermatis* was linked with a large number of TAG, FA, PE, and DG compounds (Figure 2B; Supplementary Table 2).

**Figure 3:**
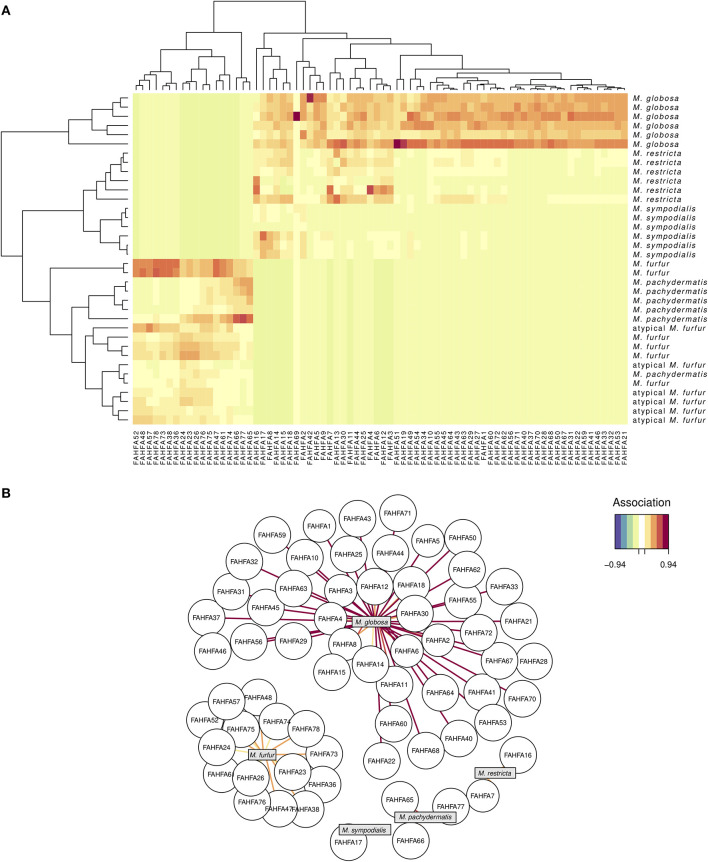
FAHFA lipid signature in five species of *Malassezia* and a putative *M. furfur* (atypical *M. furfur*) strain, six biological replicates were analyzed. A sPLS-DA (see Figure 2) barely reduced the dimensionality of 77–74 FAHFA lipids, which successfully discriminated among *M. globosa, M. restricta*, and *M. sympodialis* but not among the other studied lineages. See Figure 2 for explanation of the corresponding visualizations: the clustering heat map (above, **A**), and the correlation network (below, **B**).

Regarding the FAHFA lipid compounds, the sPLS-DA reduced the dimensionality of the original 77 to only 74 relevant compounds, represented in turn by eight PCs. Although they successfully discriminated three of the six *Malassezia* species (*M. globosa, M. restricta*, and *M. sympodialis*), there were seven misclassifications (NMC = 7) in the other *Malassezia* species (Figure 3A) and an error rate of 0.069. As expected, the AUROC values were 1.0 (*p* = 0.0013) when classifying the three former species but between 0.98 and 0.99 for the other ones. Interestingly, *M. globosa* revealed an idiosyncratic lipid profile of about 35 FAHFA compounds that were not associated with all other strains (Figure 3B). These FAHFAs belonged to previously undescribed families (Supplementary Table 3).

### *In silico* Genomic Analysis Suggested the Presence of Genes Associated With the Lipid-Biosynthesis Pathway

**Figure 4:**
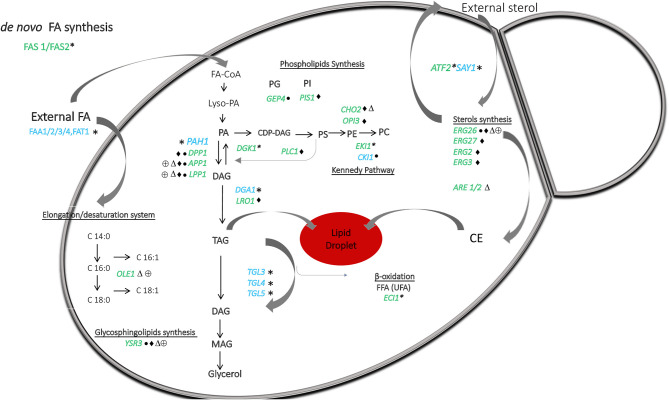
A general overview of the lipid metabolism in *Malassezia*, based on lipidomic and *in silico* genomic analysis presented in Figure 1 and Table 1. *Malassezia* species are lipid dependent due to the lack of fatty acid synthase (*FAS1/FAS2*). External sources of free fatty acids are taken up and activated by the acyl-CoA synthases *FAA1/2/3/4*. Δ9-desaturase (*OLE1*) catalyzing the conversion of saturated to unsaturated fatty acids was absent in *M. globosa* and *M. restricta*. The presence of phosphatidate phosphatase *PAH1* can replace the lack of *DPP1, LPP1*, and *APP1* to allow production of DAG from PA and to form TAG in lipid droplets. PA can normally be resynthesized from DAG by *DGK1*, but this gene was absent in all strains. Genes implicating in phospholipids synthesis involved CDP-DAG as a precursor were present in all strains. Importantly, in *M. sympodialis, PIS1* involved in PI synthesis was absent as well as *CHO2* and *OPI3*, which both are participating in the phosphatidylethanolamine N-methyltransferase pathway to form PC. *GEP4* involved in PG synthesis was absent in *M. pachydermatis*, whereas this strain did contain *CKI1* involved in PC synthesis but was absent in the other strains. *ECI1* required to degrade unsaturated FAs was absent in all strains. Genes involved in sterol and sphingolipids synthesis were present in all strains, except *ERG26* and YSR3, respectively, in *M. pachydermatis, M. sympodialis, M. globosa*, and *M. restricta*. Genes *ERG27, ERG2*, and *ERG3* were only absent in *M. sympodialis*. *ARE1/2* predicted to catalyze the acylation of ergosterol was absent in *M. globosa*. Lastly, sterol acetylation catalyzed by *ATF2* was absent in all strains. Blue letters represent the presence and in green the absence of genes denoted by *All *Malassezia* strains, • *M. pachydermatis*, ♦ *M. sympodialis*, △ *M. globosa*, and ⊕ *M. restricta*. CE represents cholesteryl ester the species.

We found the key genes involved in lipid synthesis (Table 1; Figure 4). The bioinformatic analysis suggested the presence of enzymes involved in sterol biosynthesis. Although sterols were detected, we could not differentiate among cholesterol, ergosterol, or other fungal sterols (Figure 1). *DHCR7* (7-dehydrocholesterol reductase), related to cholesterol synthesis, was present. *ERG8* (phosphomevalonate kinase), *ERG27* (sterol C-3 dehydrogenase), *ERG2* (sterol C-8 isomerases), and *ERG3* (sterol C-5 desaturases) are apparently missing in *M. sympodialis*. *ERG26* (sterol C-4 decarboxylases) homolog was present only in *M. furfur* and atypical *M. furfur* (Weete et al., 2010; Kristan and RiŽner, 2012).

TAG and CE synthesis genes were detected in the genome of all the strains (Table 1; Figure 4). *DGA1* and *LRO1* are associated with the acylation of DG to TAG and were present in almost all *Malassezia* strains. In *M. sympodialis, LRO1* is absent. Homologs of the yeast *ARE1* and *ARE2* genes that encode acyl-CoA: cholesterol acyltransferase (EC 2.3.1.26) were present in all the strains, but interestingly, these were absent in *M. globosa*. These transferases are predicted to catalyze the acylation of ergosterol, and the absence of CE in the lipidome of *M. globosa* (Figure 1) can be explained by the absence of these two genes. The phosphatidate phosphatase gene *PAH1* and the acylation gene *DGA1*, which contribute to the synthesis of TAG, were also present in all strains (Table 1; Figure 4). In contrast, the additional phosphatase genes *DPP1* (not present in *M. pachydermatis* or *M. sympodialis*), *APP1*, and *LPP1* were present in *M. furfur* (Pascual and Carman, 2013). Dgk1 (*DGK1*), a kinase related to the regulation of levels of PA, seems to be missing in all strains (Klug and Daum, 2014). The absence of this gene and the impact on *Malassezia*'s lipid metabolism require further investigation. In addition, a homolog of the alcohol O-acetyltransferase 1 gene *ATF2* was absent. This gene encodes an enzyme that, together with *SAY1* (present in all *Malassezia* strains), is part of a sterol acetylation/deacetylation cycle.

In general, homologs for enzymes involved in the glycerophospholipid synthesis were found, and an overview of the differences between the strains are summarized in Table 1 and schematically represented in Figure 4. A subset of genes involved in lipid metabolism was found to be absent in specific *Malassezia* species. In *M. sympodialis*, neither *PIS1* (phosphatidylinositol synthase), involved in the formation of PI (Klug and Daum, 2014), nor *CHO2* (phosphatidylethanolamine N-methyltransferase) or *OPI3* (CDP-diacylglycerol-serine O-phosphatidyltransferase), which catalyze the reactions to the formation of PC, were found. This latter gene was also missing in *M. globosa*. *PLC1* encoding phospholipase C was missing in *M. sympodialis*. Homologs for enzymes related to the synthesis of PE via the Kennedy pathway, such as *EKI1* (ethanolamine kinase), were apparently absent in all strains. The choline kinase gene *CKI1*, required for PC synthesis, was present only in *M. pachydermatis* (Klug and Daum, 2014). An important enzyme encoded by (*GEP4*) involved in the dephosphorylation of PGP to PG was apparently not present in *M. pachydermatis* (Figure 4) (Henry et al., 2012).

The glycosphingolipid synthesis genes were present, except *YSR3* (dihydrosphingosine 1-phosphate phosphatase), which was present only in *M. furfur* and atypical *M. furfur*; however, a paralog *LCB3* was present in all strains (Table 1; Figure 4).

Lastly, homologous genes for enzymes required to degrade unsaturated FAs, such as *ECI1*, were absent in all *Malassezia* strains (Figure 4), as was reported previously (Gordon James et al., 2013). In contrast, *SPS19* (2,4-dienoyl-CoA reductase) and *DCI1* (delta (3,5)-delta (2,4)-dienoyl-CoA isomerase) were detected in our analysis. *FAD2/3* Δ12-desaturase/ω3-desaturase, involved in the synthesis of PUFAs such as linoleic acid (LA, C18:2n-6) and α-linolenic acid (ALA, C18:3n-3), was present (Table 1; Figure 4) (Leonard et al., 2004).

## Discussion

*Malassezia* yeast lacks *de novo* FA synthesis, and it is therefore lipophilic. Limited information is present about the lipid metabolism in these species as well as the lipidic components produced. Here, we present the results of lipidomic and *in silico* genomic analysis of six strains of *Malassezia* (*M. globosa, M. sympodialis, M. restricta, M. furfur, M. pachydermatis*, and atypical *M. furfur*). The data reveal novel insights and are of importance to understand lipid metabolism in this yeast.

Lipid content based on the currently described analysis allowed us to discriminate the *Malassezia* strains because the phylogenetic relations were maintained among the species during the examination of their lipid composition. In this study, we determined that the most common lipids of these strains are TAG, sterols, diglycerides, FAs, phosphatidylcholine, phosphatidylethanolamine, ceramides, sphingomyelin, acylcarnitine, and lysophospholipids. The lipid composition was similar to that of *S. cerevisiae* (Kohlwein, 2017). Furthermore, we cannot exclude the possibility that other lipid classes are present but were left undetected due to the currently used methods of extraction on stationary grown cells (e.g., PI would be subject to future research). Lipidomic analysis of mDixon medium itself is also necessary to clarify whether specific association of lipids from the medium does occur.

Importantly, we could not differentiate among sterols, and further sterol profiling is required to determine which species are actually produced. Sterols could also be taken up as indicated in Figure 4 and used in the production of CE stored in lipid droplets. We did, however, detect the apparent presence of all genes encoding sterol synthesis enzymes. It would be important to differentiate among sterol species due to the fact that sterols others than ergosterol, regarded as the “fungal sterol,” have been described. Ergosterol was considered the unique fungal sterol. However, other sterol species, such as lanosterol, brassicasterol, 24-ethyl cholesterol, 24-methyl cholesterol, and cholesterol, are characteristic for some fungal groups included in the phyla Chytridiomycota and Mucoromycota. These sterols may also be present in the Dikarya (Weete et al., 2010). Particularly, the homolog of *DHCR7* (7-dehydrocholesterol reductase) that is related to cholesterol synthesis was found in the *Malassezia* genomes. *Pneumocystis jirovecii*, an important human pathogen, is characterized by the presence of cholesterol, and previous studies have suggested that it contributes to the flexibility of the membrane in the trophic form of these fungi (Ma et al., 2016). Among the phylum Basidiomycota, rust fungi contain intermediates in the formation of 24-ethyl cholesterol (Weete et al., 2010). Further analyses are required to corroborate the presence of the sterol species in *Malassezia* species.

TAG and CE are neutral lipids produced from FA and sterols, respectively, to avoid possible toxicity due to an excess of these compounds in the cell (Klug and Daum, 2014). A relatively high amount of neutral lipids was detected in some of the *Malassezia* species. In biological membranes, the phospholipid-to-cholesterol ratio cannot exceed 2:1; we believe that the ratio of phospholipids is higher and that the relatively high amount of neutral lipids might be due to the currently used methods of analysis. In the future, different methods of extraction are required to clarify this issue. It should, however, be mentioned that *Malassezia* species were reported to have a lipid-rich cell wall (Hechemy and Vanderwyk, 1968; Thompson and Colvin, 1970) and even a lipid-like capsular layer (Mittag, 1995) that modulates immune responses (Thomas et al., 2008), and the possibility that some of the neutral lipids are derived from this source requires further investigation. TAG were present in different concentrations among the strains with a wide range of molecular species, and some of them showed a positive association with *M. pachydermatis* that enables the differentiation of this strain from the others. We identified genes related to TAG synthesis. Particularly, *LRO1*, which is associated with the acylation of DG to TAG, seemed to be absent in *M. sympodialis* (Klug and Daum, 2014). However, *DGA1*, which has the same function, was present in this strain; thus, the possible absence of *LRO1* might not affect the TAG synthesis (Klug and Daum, 2014).

We found a low content of CEs in *M. furfur*, atypical *M. furfur*, and *M. pachydermatis* and undetectable traces of these components in *M. globosa, M. restricta*, and *M. sympodialis*. On the other hand, *ARE1* and *ARE2* genes are transferases predicted to catalyze the acylation of ergosterol to CE and were present in all strains except *M. globosa*. The absence of these genes in *M. globosa* may explain why CE was undetected in this strain, but it cannot explain the absence in *M. sympodialis* and *M. restricta* unless these genes are not expressed. Expression analysis is required to corroborate differences in the functionality of *ARE1/ARE2* in *Malassezia* strains. These differences could also be due to possible inhibition of CE synthesis by oleic acid due to competitive inhibition of *Are2p* by free oleate, as has been described for *S. cerevisiae*; however, all strains were grown in the same medium (Connerth et al., 2010; Grillitsch et al., 2011).

The mechanism used by the cell to avoid toxic effects due to excess of sterols involves HMG-CoA reductase (HMGR), a conserved enzyme in eukaryotes, and the acetylation/deacetylation cycle performed by *ATF2/SAY1* (Burg and Espenshade, 2011; Klug and Daum, 2014). Here, we did not detect homologs to *ATF2*, but a homolog of *SAY1* was shown to be present in all strains. The absence of *ATF2* would imply that this detoxifying mechanism is not operative in *Malassezia* or that these strains contain a different enzyme. The presence of organelles such as lipid droplets may contribute to the detoxification mechanisms (Celis Ramírez, 2017). Why high levels of sterols are tolerated in some of the species remains to be determined.

Phospholipids are structural components of membranes and play many essential roles in cell biology, like membrane trafficking, membrane identity, and anchoring of membrane proteins, and also serve as signaling molecules and as precursors of signaling molecules. We found differences in the content of PC and PE, which were detected in higher concentrations than PS, phosphatidylglycerol (PG), and cardiolipin. CDP-DAG and Kennedy pathways can synthesize PE and PC (Klug and Daum, 2014). Genes associated with CDP-DAG were present in all strains, but important genes in the Kennedy pathways were not (*EKI1* was absent in all strains, and *CKI1* was present only in *M. pachydermatis)*. This probably means that *Malassezia* strains can synthesize PE and PC only using CDP-DAG and that *M. pachydermatis* can synthesize PC also via the Kennedy pathway. *PLC1* encodes phospholipase C, forming DG, inositol, and G3P (glycerol-3-phosphate), which can serve again as precursors for phospholipid synthesis (Henry et al., 2012). This is missing in *M. sympodialis* and may be related to differences observed in the metabolism of this species. However, further analyses are required to confirm this. GEP4p is involved in the dephosphorylation of PGP to PG and is apparently not present in *M. pachydermatis* (Henry et al., 2012), and it may be the reason why this lipid species was in fact not detected in our analysis.

Homologs to several genes (among others, *ERG26, ERG27, ERG2, ERG3, PIS1, CHO2, OPI3, PLC1, CKI1, EKI1*, and *GEP4*; see Table 1 for a complete overview) were not identified in some strains, and *M. sympodialis* and *M. globose*, in particular, were missing most homologs. However, we cannot exclude the presence of genes with similar functions that might have escaped detection due to very limited similarity.

PUFAs are important structural components that confer membrane fluidity and selective permeability (Leonard et al., 2004). Arachidonic acid (ARA; 20:4) and docosahexaenoic acid (DHA; 22:6) were detected in our analysis. These FAs are not produced by *S. cerevisiae*, which mainly produces saturated and monounsaturated FAs of 16- and 18-carbon compounds because it contains only one FA desaturase, a Δ9-desaturase (OLE1) (Uemura, 2012). However, bifunctional D12/D15-FADs can desaturate D9-UFAs to PUFAs, and these have been detected in different species belonging to Basidiomycota and Ascomycota (Buček et al., 2014). A previous study predicted the metabolism of ARA in atypical *M. furfur*, suggesting its role as a precursor of eicosanoids (Triana et al., 2017). We were able to detect the enzymes involved in the biosynthesis of these FAs.

The recently discovered class of FAHFA lipids (Yore et al., 2014) was detected in *Malassezia* species with, additionally, a high variety in the composition of their acyl chains. It is noteworthy that some of these lipids have not been reported previously (Liberati-Cizmek et al., 2019). These lipids might provide anti-inflammatory properties, but further studies should provide insights into their role in fungal biology and host–pathogen interactions (Zhu et al., 2018).

Betaine lipid diacylglycerol-trimethyl-homoserine (DGTS) is an analog of phosphatidylcholine (PtdCho), which is synthesized by many soil bacteria, green plants, chromophytes, fungi, and amoebae (Sohlenkamp and Geiger, 2016). In the human fungal pathogens, *Candida albicans* and *Cryptococcus neoformans* phosphate starvation induces the replacement of phosphatidylcholine with betaine lipid, and this event was related to fungal virulence during host interaction (Naik et al., 2017; Lev et al., 2019). DGTS was also detected in *M. globosa, M. sympodialis*, and *M. restricta*. Some evidence suggests reciprocity between PC and DGTS content, meaning that, in most cases, in which PC is a major lipid, no DGTS is detected and vice versa. However, this was not the case in this study. In some algae, PC and DGTS accumulate at the same time (Sohlenkamp and Geiger, 2016). The role of DGTS in *Malassezia* species requires further analysis.

The lipidomic analysis of the *Malassezia* species described in this paper is in the context of growth to the stationary phase in mDixon broth. This is a rich medium that contains the lipid-containing components Ox bile, peptone, malt extract, oleic acid (>78% pure), and Tween 40 (>90% pure). Ox bile is a complex lipid mixture containing bile salts and bilirubin cholesterol, FAs, and lecithins, which are a mixture of different phospholipids, glycolipids, or TAG (Hall and Guyton, 2011). Peptone is a protein hydrolysate that contains small amounts (~0.6%) of lipids (Klompong et al., 2009). Malt extract is prepared by extracting the soluble products from sprouted grain and might contain lipids, but to our knowledge, a detailed analysis has not been presented.

There is a possibility that some of the lipidic components present in the mDixon associate with yeast cells and were detected in our analysis. The relative amount of the different lipid species we detected in the six different *Malassezia* species (Figure 1) do, however, vary considerably between these species. These rules out the possibility that similar amounts of the medium lipids were simply carried over with these six species because they all were cultured in the same medium. Whether differential association of specific lipid species to cells occurs remains to be determined, but it would be very remarkable and might suggest that different *Malassezia* species have a preference for accumulating certain lipid sources from the medium. A lipidomic analysis of the medium is required to confirm that preferential association does occur. To eliminate the problem of carryover of lipids, synthetic media with pure lipids as a source should be used, but such a medium has not yet been developed. Furthermore, more work is required to link the current lipid analysis to lipidomic analysis of *Malassezia* species grown with culturing methods that resemble the *in vivo* conditions, which remains a major challenge. In addition, the role of gene products and their importance in the biosynthetic pathways require further studies, such as knockouts, mutant genes as well as studies with stable isotope labeled FAs coupled with lipidomic analysis to unravel these routes.

Taken together, our data provide a general overview of the lipid composition and metabolism in six *Malassezia* strains. This study contributes to the knowledge in this genus and provides fundamental information with which future studies can advance comprehensive knowledge of the role of lipids in the life cycle of *Malassezia* yeast as commensal and pathogen.

## Data Availability Statement

All datasets generated for this study are included in the article/Supplementary Material.

## Author Contributions

AC, AA, AB, and SR contributed to the design of the work. AC performed the experiments. AC, AA, JC, LM-C, JA-M, and HC wrote the manuscript. AC, HC, SR, and AB made revisions. All authors were involved in the analysis and interpretation of data. All authors approved the version to be published and agreed to be accountable for all aspects of the work.

## Conflict of Interest

The authors declare that the research was conducted in the absence of any commercial or financial relationships that could be construed as a potential conflict of interest.

## Footnotes

^1^http://www.R-project.org/

## Supplementary Material

Supplementary Table 1 Raw data with concentration (%mol) of lipid species detected in *Malassezia* strains.

Supplementary Table 2 Class of lipid species detected by UHPLC/MS.

Supplementary Table 3 FAHFA species detected by UHPLC/MS. ^*^Abbreviations were adapted from: Kolar et al. (2019), Liberati-Cizmek et al. (2019), Balas et al. (2018), Zhu et al. (2018), Hu et al. (2018), and Kuda et al. (2016).
