# Supplementary material for: Analysis of Malassezia Lipidome Disclosed Differences Among the Species and Reveals Presence of Unusual Yeast Lipids
Source: Front Cell Infect Microbiol. 2020 Jul 15;10:338. doi: 10.3389/fcimb.2020.00338 (PMC7374198; doi:10.3389/fcimb.2020.00338)
Supplement: Supplementary Table 2 — Class of lipid species detected by UHPLC/MS. [file Table_2.docx]

**Table S2.** Class of lipid species detected by UHPLC/MS.

| **Compound Identification** | **Compound Type** | **Compound** |
| --- | --- | --- |
| AC1 | Acylcarnitine | Acylcarnitine (10:0) |
| AC2 |  | Acylcarnitine (12:0) |
| AC3 |  | Acylcarnitine (16:0) |
| AC4 |  | Acylcarnitine (10:1) |
| AC5 |  | Acylcarnitine (12:1) |
| AC6 |  | Acylcarnitine (14:1) |
| AC7 |  | Acylcarnitine (14:2) |
| AC8 |  | Acylcarnitine (18:1) |
| CE1 | Cholesteryl ester  (Steryl ester)* | CE (18:2) |
| CE2 |  | CE (18:3) |
| CE3 |  | CE (20:3) |
| CE4 |  | CE (20:4) |
| CE5 |  | CE (20:5) |
| CE6 |  | CE (22:6) |
| CER1 | Ceramide | Ceramide (d34:1) |
| CER2 |  | Ceramide (d36:1) |
| CER3 |  | Ceramide (d38:1) |
| CER4 |  | Ceramide (d38:1) |
| CER5 |  | Ceramide (d41:0) |
| CER6 |  | Ceramide (d41:1) |
| CER7 |  | Ceramide (d42:1) |
| CER8 |  | Ceramide (d18:1/23:0) |
| CER9 |  | Ceramide (d32:1) |
| CER10 |  | Ceramide (d34:1) |
| CER11 |  | Ceramide (d34:0) |
| CER12 |  | Ceramide (d34:2) |
| CER13 |  | Ceramide (d39:1) |
| CER14 |  | Ceramide (d40:0) |
| CER15 |  | Ceramide (d40:2)A |
| CER16 |  | Ceramide (d40:2)B |
| CER17 |  | Ceramide (d42:0) |
| CER18 |  | Ceramide (d42:2) |
| CER19 |  | Ceramide (d42:2)A |
| CER20 |  | Ceramide (d36:1) |
| CER21 |  | Ceramide (d43:1) |
| DG1 | Diacylglycerol | DG (32:0) |
| DG2 |  | DG (32:1) |
| DG3 |  | DG (34:1) |
| DG4 |  | DG (34:2) |
| DG5 |  | DG (36:2) |
| DG6 |  | DG (36:3) |
| DG7 |  | DG (38:5) |
| DG8 |  | DG (34:3) |
| DG9 |  | DG (36:1) |
| DG10 |  | DG (36:4)A |
| DG11 |  | DG (36:4)B |
| DG12 |  | DG (36:5) |
| DG13 |  | DG (36:6) |
| DG14 |  | DG (38:6) |
| DGTS1 | Diacylglyceryltrimethylhomoserine | DGTS(34:1);DGTS(16:0-18:1) |
| DGTS2 |  | DGTS(34:2);DGTS(16:1-18:1) |
| DGTS3 |  | DGTS(34:3) |
| DGTS4 |  | DGTS(36:3);DGTS(18:1-18:2) |
| ERG | Ergosterol | Ergosterol |
| FA1 | Fatty acid | FA(11:0) (undecylic acid) |
| FA2 |  | FA(12:0) (lauric acid) |
| FA3 |  | FA(13:0) (tridecylic acid) |
| FA4 |  | FA(14:0) (myristic acid) |
| FA5 |  | FA(14:1) (physeteric acid) |
| FA6 |  | FA(15:0) (pentadecylic acid) |
| FA7 |  | FA(15:1) (pentadecenoic acid) |
| FA8 |  | FA(16:0) (palmitic acid) |
| FA9 |  | FA(16:1) (palmitoleic acid) |
| FA10 |  | FA(16:2) (hexadecadienoic acid) |
| FA11 |  | FA(16:3) (hexadecatrienoic acid) |
| FA12 |  | FA(16:4) (hexadecatetraenoic acid) |
| FA13 |  | FA(17:0) (margaric acid) |
| FA14 |  | FA(17:1) (heptadecenoic acid) |
| FA15 |  | FA(17:2) (heptadecadienoic acid) |
| FA16 |  | FA(18:0) (stearic acid) |
| FA17 |  | FA(18:1) (oleic acid) |
| FA18 |  | FA(18:2) (linoleic acid) |
| FA19 |  | FA(18:3) (linolenic acid) |
| FA20 |  | FA(18:4) (stearidonic acid) |
| FA21 |  | FA(19:0) (nonadecanoic acid) |
| FA22 |  | FA(19:1) (nonadecenoic acid) |
| FA23 |  | FA(19:2) (nonadecadienoic acid) |
| FA24 |  | FA(19:3) (nonadecatrienoic acid) |
| FA25 |  | FA(20:0) (arachidic acid) |
| FA26 |  | FA(20:1) (eicosenoic acid) |
| FA27 |  | FA(20:2) (eicosadienoic acid) |
| FA28 |  | FA(20:3) (eicosatrienoic acid) |
| FA29 |  | FA(20:3) (homo-gamma-linolenic acid) |
| FA30 |  | FA(20:4) (arachidonic acid) |
| FA31 |  | FA(20:5) (eicosapentaenoic acid) |
| FA32 |  | FA(21:0) (heneicosanoic acid) |
| FA33 |  | FA(21:1) (heneicosenoic acid) |
| FA34 |  | FA(21:2) (heneicodienoic acid) |
| FA35 |  | FA(21:4) (heneicotetraenoic acid) |
| FA36 |  | FA(21:5) (heneicopentaenoic acid) |
| FA37 |  | FA(22:0) (behenic acid) |
| FA38 |  | FA(22:1) (erucic acid) |
| FA39 |  | FA(22:2) (docosadienoic acid) |
| FA40 |  | FA(22:4) (docosatetraenoic acid) |
| FA41 |  | FA(22:5) (docosapentaenoic acid) |
| FA42 |  | FA(22:6)(docosahexaenoic acid) |
| FA43 |  | FA(24:0) (lignoceric acid) |
| FA44 |  | FA(24:1) (nervonic acid) |
| FA45 |  | FA(26:0) (cerotic acid) |
| FA46 |  | FA(28:0) (montanic acid) |
| FA47 |  | FA(22:3) (docosatrienoic acid) |
| FAHFA1 | Fatty acid ester of hydroxyl fatty acid | FAHFA(18:0);FAHFA(11:0/7:0) |
| FAHFA2 |  | FAHFA(23:1);FAHFA(18:1/5:0) |
| FAHFA3 |  | FAHFA(24:0);FAHFA(16:0/8:0) |
| FAHFA4 |  | FAHFA(24:1);FAHFA(16:1/8:0) |
| FAHFA5 |  | FAHFA(24:1);FAHFA(18:1/6:0) |
| FAHFA6 |  | FAHFA(25:0);FAHFA(16:0/9:0) |
| FAHFA7 |  | FAHFA(25:0);FAHFA(18:0/7:0) |
| FAHFA8 |  | FAHFA(25:1);FAHFA(18:1/7:0) |
| FAHFA9 |  | FAHFA(25:2);FAHFA(18:2/7:0) |
| FAHFA10 |  | FAHFA(26:0);FAHFA(10:0/16:0) |
| FAHFA11 |  | FAHFA(26:0);FAHFA(16:0/10:0) |
| FAHFA12 |  | FAHFA(26:0);FAHFA(18:0/8:0) |
| FAHFA13 |  | FAHFA(26:1);FAHFA(10:0/16:1) |
| FAHFA14 |  | FAHFA(26:1);FAHFA(18:1/8:0) |
| FAHFA15 |  | FAHFA(26:2);FAHFA(18:2/8:0) |
| FAHFA16 |  | FAHFA(27:0);FAHFA(18:0/9:0) |
| FAHFA17 |  | FAHFA(27:1);FAHFA(18:1/9:0) |
| FAHFA18 |  | FAHFA(27:2);FAHFA(18:2/9:0) |
| FAHFA19 |  | FAHFA(28:0);FAHFA(12:0/16:0) |
| FAHFA20 |  | FAHFA(28:0);FAHFA(18:0/10:0) |
| FAHFA21 |  | FAHFA(28:1);FAHFA(10:0/18:1) |
| FAHFA22 |  | FAHFA(28:1);FAHFA(12:0/16:1) |
| FAHFA23 |  | FAHFA(36:2);FAHFA(18:1/18:1) |
| FAHFA24 |  | FAHFA(36:3);FAHFA(18:1/18:2) |
| FAHFA25 |  | FAHFA(36:4);FAHFA(18:1/18:3) |
| FAHFA26 |  | FAHFA(36:4);FAHFA(18:2/18:2) |
| FAHFA27 |  | FAHFA(36:5);FAHFA(18:3/18:2) |
| FAHFA28 |  | FAHFA(36:6);FAHFA(18:3/18:3) |
| FAHFA29 |  | FAHFA(38:2);FAHFA(18:1/20:1) |
| FAHFA30 |  | FAHFA(38:4);FAHFA(18:2/20:2) |
| FAHFA31 |  | FAHFA(38:4);FAHFA(20:3/18:1) |
| FAHFA32 |  | FAHFA(38:5);FAHFA(20:4/18:1) |
| FAHFA33 |  | FAHFA(30:3);FAHFA(14:1/16:2) |
| FAHFA34 |  | FAHFA(31:0);FAHFA(15:0/16:0) |
| FAHFA35 |  | FAHFA(31:1);FAHFA(15:0/16:1) |
| FAHFA36 |  | FAHFA(32:0);FAHFA(16:0/16:0) |
| FAHFA37 |  | FAHFA(32:1);FAHFA(14:0/18:1) |
| FAHFA38 |  | FAHFA(32:1);FAHFA(16:1/16:0) |
| FAHFA39 |  | FAHFA(32:2);FAHFA(14:1/18:1) |
| FAHFA40 |  | FAHFA(32:2);FAHFA(16:0/16:2) |
| FAHFA41 |  | FAHFA(32:2);FAHFA(16:1/16:1) |
| FAHFA42 |  | FAHFA(32:2);FAHFA(18:1/14:1) |
| FAHFA43 |  | FAHFA(32:3);FAHFA(16:1/16:2) |
| FAHFA44 |  | FAHFA(32:4);FAHFA(16:3/16:1) |
| FAHFA45 |  | FAHFA(32:5);FAHFA(16:3/16:2) |
| FAHFA46 |  | FAHFA(32:5);FAHFA(16:4/16:1) |
| FAHFA47 |  | FAHFA(33:0);FAHFA(16:0/17:0) |
| FAHFA48 |  | FAHFA(33:1);FAHFA(17:1/16:0) |
| FAHFA49 |  | FAHFA(33:2);FAHFA(18:1/15:1) |
| FAHFA50 |  | FAHFA(33:3);FAHFA(17:1/16:2) |
| FAHFA51 |  | FAHFA(34:0);FAHFA(18:0/16:0) |
| FAHFA52 |  | FAHFA(34:1);FAHFA(18:1/16:0) |
| FAHFA53 |  | FAHFA(34:2);FAHFA(16:1/18:1) |
| FAHFA54 |  | FAHFA(34:2);FAHFA(18:2/16:0) |
| FAHFA55 |  | FAHFA(34:3);FAHFA(16:1/18:2) |
| FAHFA56 |  | FAHFA(34:3);FAHFA(18:1/16:2) |
| FAHFA57 |  | FAHFA(34:3);FAHFA(18:3/16:0) |
| FAHFA58 |  | FAHFA(34:4);FAHFA(18:2/16:2) |
| FAHFA59 |  | FAHFA(34:5);FAHFA(18:3/16:2) |
| FAHFA60 |  | FAHFA(35:1);FAHFA(17:0/18:1) |
| FAHFA61 |  | FAHFA(35:1);FAHFA(18:1/17:0) |
| FAHFA62 |  | FAHFA(35:2);FAHFA(17:1/18:1) |
| FAHFA63 |  | FAHFA(35:2);FAHFA(18:1/17:1) |
| FAHFA64 |  | FAHFA(35:3);FAHFA(18:2/17:1) |
| FAHFA65 |  | FAHFA(36:0);FAHFA(18:0/18:0) |
| FAHFA66 |  | FAHFA(36:1);FAHFA(18:1/18:0) |
| FAHFA67 |  | FAHFA(40:4);FAHFA(16:0/24:4) |
| FAHFA68 |  | FAHFA(40:7);FAHFA(22:6/18:1) |
| FAHFA69 |  | FAHFA(42:1);FAHFA(18:1/24:0) |
| FAHFA70 |  | FAHFA(42:5);FAHFA(18:1/24:4) |
| FAHFA71 |  | FAHFA(42:6);FAHFA(18:2/24:4) |
| FAHFA72 |  | FAHFA(44:1);FAHFA(26:0/18:1) |
| FAHFA73 |  | FAHFA(30:0);FAHFA(14:0/16:0) |
| FAHFA74 |  | FAHFA(34:0);FAHFA(16:0/18:0) |
| FAHFA75 |  | FAHFA(34:1);FAHFA(16:0/18:1) |
| FAHFA76 |  | FAHFA(36:3);FAHFA(18:2/18:1) |
| FAHFA77 |  | FAHFA(36:4);FAHFA(20:4/16:0) |
| GCER1 | Glucosylceramide | GlcCer(d42:1) |
| GCER2 |  | GlcCer(d40:1) |
| GCER3 |  | GlcCer(d42:2) |
| LPC1 | Lysophosphatidylcholine | LPC(14:0) |
| LPC2 |  | LPC(15:0) |
| LPC3 |  | LPC(16:0) |
| LPC4 |  | LPC(16:1) |
| LPC5 |  | LPC(17:1) |
| LPC6 |  | LPC(18:0) |
| LPC7 |  | LPC(18:0)A |
| LPC8 |  | LPC(18:0)B |
| LPC9 |  | LPC(18:1) |
| LPC10 |  | LPC(18:2) |
| LPC11 |  | LPC(18:3) |
| LPC12 |  | LPC(20:1) |
| LPC13 |  | LPC(20:3) |
| LPC14 |  | LPC(20:4) |
| LPC15 |  | LPC(20:5) |
| LPC16 |  | LPC(20:2) |
| LPE1 | Lysophosphatidylethanolamine | LPE(16:0) |
| LPE2 |  | LPE(16:1) |
| LPE3 |  | LPE(18:0) |
| LPE4 |  | LPE(18:2) |
| LPI1 | Lysophosphatidylinositol | LPI(16:0) |
| LPI2 |  | LPI(18:0) |
| LPI3 |  | LPI(18:1) |
| PA1 | Phosphatidic acid | PA(34:1);PA(16:0-18:1) |
| PA2 |  | PA(36:2);PA(18:1-18:1) |
| PA3 |  | PA(36:3);PA(18:1-18:2) |
| PA4 |  | PA(36:4);PA(18:2-18:2) |
| PC1 | Phosphatidylcholine | PC(p-44:5)orPC(o-44:6) |
| PC2 |  | PC(31:0) |
| PC3 |  | PC(32:0) |
| PC4 |  | PC(32:1) |
| PC5 |  | PC(32:2) |
| PC6 |  | PC(33:0) |
| PC7 |  | PC(33:1) |
| PC8 |  | PC(34:0) |
| PC9 |  | PC(34:1) |
| PC10 |  | PC(34:2) |
| PC11 |  | PC(34:3) |
| PC12 |  | PC(34:4) |
| PC13 |  | PC(35:1) |
| PC14 |  | PC(35:2) |
| PC15 |  | PC(35:3) |
| PC16 |  | PC(35:4) |
| PC17 |  | PC(36:1) |
| PC18 |  | PC(36:2) |
| PC19 |  | PC(36:3)A |
| PC20 |  | PC(36:3)B |
| PC21 |  | PC(36:4)A |
| PC22 |  | PC(36:4)B |
| PC23 |  | PC(36:5)A |
| PC24 |  | PC(37:2) |
| PC25 |  | PC(37:3) |
| PC26 |  | PC(38:2) |
| PC27 |  | PC(38:3) |
| PC28 |  | PC(38:4)A |
| PC29 |  | PC(38:4)B |
| PC30 |  | PC(38:5)A |
| PC31 |  | PC(38:5)B |
| PC32 |  | PC(40:4) |
| PC33 |  | PC(40:8) |
| PC34 |  | PC(o-32:0) |
| PC35 |  | PC(o-34:0) |
| PC36 |  | PC(p-32:0)orPC(o-32:1) |
| PC37 |  | PC(p-34:1)orPC(o-34:2)A |
| PC38 |  | PC(p-34:2)orPC(o-34:3) |
| PC39 |  | PC(p-36:3)orPC(o-36:4) |
| PC40 |  | PC(p-36:4)orPC(o-36:5) |
| PC41 |  | PC(p-38:3)orPC(o-38:4) |
| PC42 |  | PC(p-38:4)orPC(o-38:5)A |
| PC43 |  | PC(p-40:6)orPC(o-40:7)A |
| PC44 |  | PC(p-40:7)orPC(o-40:8) |
| PC45 |  | PC(p-42:3)orPC(o-42:4) |
| PC46 |  | PC(p-42:4)orPC(o-42:5) |
| PC47 |  | PC(p-42:5)orPC(o-42:6) |
| PC48 |  | PC(38:3);PC(14:1/24:2) |
| PC49 |  | PC(34:3)A |
| PC50 |  | PC(34:3)B |
| PC51 |  | PC(34:3)C |
| PC52 |  | PC(35:2)A |
| PC53 |  | PC(35:2)B |
| PC54 |  | PC(36:4)C |
| PC55 |  | PC(37:4) |
| PC56 |  | PC(38:1) |
| PC57 |  | PC(38:4)C |
| PC58 |  | PC(38:6)C |
| PC59 |  | PC(p-38:4)/PC(o-38:5)B |
| PC60 |  | PC(p-38:6)/PC(o-38:7) |
| PC61 |  | PC(p-40:6)/PC(o-40:7)B |
| PE1 | Phosphatidylethanolamine | PE(34:1) |
| PE2 |  | PE(34:2) |
| PE3 |  | PE(35:1);PE(17:0-18:1) |
| PE4 |  | PE(35:2);PE(17:1-18:1) |
| PE5 |  | PE(36:1) |
| PE6 |  | PE(36:2) |
| PE7 |  | PE(36:3) |
| PE8 |  | PE(36:4) |
| PE9 |  | PE(38:2) |
| PE10 |  | PE(38:2);PE(18:1-20:1) |
| PE11 |  | PE(p-34:2)orPE(o-34:3) |
| PE12 |  | PE(p-36:2)orPE(o-36:3) |
| PE13 |  | PE(p-36:4)orPE(o-36:5) |
| PE14 |  | PE(p-38:4)orPE(o-38:5) |
| PE15 |  | PE(34:1);PE(16:0/18:1) |
| PE16 |  | PE(36:2);PE(18:1/18:1) |
| PE17 |  | PE(36:3);PE(18:1/18:2) |
| PE18 |  | PE(38:4) |
| PE19 |  | PE(38:6) |
| PG1 | Phosphatidylglycerol | PG(34:1);PG(16:0-18:1) |
| PG2 |  | PG(34:2);PG(16:0-18:2) |
| PG3 |  | PG(36:1);PG(18:0-18:1) |
| PG4 |  | PG(36:2);PG(18:1-18:1) |
| PG5 |  | PG(36:3);PG(18:1-18:2) |
| SM1 | Sphingomyelin | SM(d32:1) |
| SM2 |  | SM(d32:2) |
| SM3 |  | SM(d33:1) |
| SM4 |  | SM(d34:0) |
| SM5 |  | SM(d34:1) |
| SM6 |  | SM(d34:2) |
| SM7 |  | SM(d36:0) |
| SM8 |  | SM(d36:1) |
| SM9 |  | SM(d36:2) |
| SM10 |  | SM(d36:3) |
| SM11 |  | SM(d38:2) |
| SM12 |  | SM(d39:1) |
| SM13 |  | SM(d40:1) |
| SM14 |  | SM(d40:2)A |
| SM15 |  | SM(d41:1) |
| SM16 |  | SM(d42:1) |
| SM17 |  | SM(d42:2)A |
| SM18 |  | SM(d42:3) |
| SM19 |  | SM(d43:2)A |
| SM20 |  | SM(d40:0) |
| SM21 |  | SM(d43:1) |
| TAG1 | Triacylglycerol | TAG(44:2) |
| TAG2 |  | TAG(46:2) |
| TAG3 |  | TAG(46:3)A |
| TAG4 |  | TAG(46:3)B |
| TAG5 |  | TAG(47:1) |
| TAG6 |  | TAG(48:1) |
| TAG7 |  | TAG(48:2) |
| TAG8 |  | TAG(48:3) |
| TAG9 |  | TAG(48:4)A |
| TAG10 |  | TAG(48:4)B |
| TAG11 |  | TAG(48:5) |
| TAG12 |  | TAG(48:6) |
| TAG13 |  | TAG(49:1) |
| TAG14 |  | TAG(49:1);TAG(15:0-16:0-17:1); |
| TAG15 |  | TAG(49:2) |
| TAG16 |  | TAG(49:3) |
| TAG17 |  | TAG(50:0) |
| TAG18 |  | TAG(50:1) |
| TAG19 |  | TAG(50:2) |
| TAG20 |  | TAG(50:3)A |
| TAG21 |  | TAG(50:3)B |
| TAG22 |  | TAG(50:4) |
| TAG23 |  | TAG(50:5) |
| TAG24 |  | TAG(50:6) |
| TAG25 |  | TAG(51:1) |
| TAG26 |  | TAG(51:2) |
| TAG27 |  | TAG(51:2);TAG(16:1-17:1-18:0) |
| TAG28 |  | TAG(51:3) |
| TAG29 |  | TAG(51:4) |
| TAG30 |  | TAG(51:5) |
| TAG31 |  | TAG(52:0) |
| TAG32 |  | TAG(52:1) |
| TAG33 |  | TAG(52:1);TAG(14:0-18:1-20:0) |
| TAG34 |  | TAG(52:2) |
| TAG35 |  | TAG(52:2);TAG(16:0-16:0-20:2) |
| TAG36 |  | TAG(52:2);TAG(16:0-18:1-18:1) |
| TAG37 |  | TAG(52:3) |
| TAG38 |  | TAG(52:4) |
| TAG39 |  | TAG(52:5) |
| TAG40 |  | TAG(52:6) |
| TAG41 |  | TAG(52:6);TAG(16:0-18:3-18:3) |
| TAG42 |  | TAG(53:1) |
| TAG43 |  | TAG(53:2) |
| TAG44 |  | TAG(53:3) |
| TAG45 |  | TAG(53:4) |
| TAG46 |  | TAG(53:5) |
| TAG47 |  | TAG(54:1) |
| TAG48 |  | TAG(54:2) |
| TAG49 |  | TAG(54:3) |
| TAG50 |  | TAG(54:3);TAG(18:0-18:1-18:2) |
| TAG51 |  | TAG(54:4) |
| TAG52 |  | TAG(54:5)A |
| TAG53 |  | TAG(54:5)B |
| TAG54 |  | TAG(54:6)A |
| TAG55 |  | TAG(54:6)C |
| TAG56 |  | TAG(54:7)A |
| TAG57 |  | TAG(54:9);TAG(18:3-18:3-18:3) |
| TAG58 |  | TAG(55:1) |
| TAG59 |  | TAG(55:2) |
| TAG60 |  | TAG(55:3) |
| TAG61 |  | TAG(55:4);TAG(18:1-18:2-19:1) |
| TAG62 |  | TAG(56:1) |
| TAG63 |  | TAG(56:2) |
| TAG64 |  | TAG(56:3) |
| TAG65 |  | TAG(56:4) |
| TAG66 |  | TAG(56:5)A |
| TAG67 |  | TAG(56:5)B |
| TAG68 |  | TAG(56:5)C |
| TAG69 |  | TAG(56:6) |
| TAG70 |  | TAG(56:7)A |
| TAG71 |  | TAG(57:1) |
| TAG72 |  | TAG(57:2) |
| TAG73 |  | TAG(57:3);TAG(18:1-19:1-20:1) |
| TAG74 |  | TAG(58:1) |
| TAG75 |  | TAG(58:2) |
| TAG76 |  | TAG(58:3) |
| TAG77 |  | TAG(58:4) |
| TAG78 |  | TAG(58:5) |
| TAG79 |  | TAG(58:6) |
| TAG80 |  | TAG(58:7);TAG(18:1-18:1-22:5) |
| TAG81 |  | TAG(59:2) |
| TAG82 |  | TAG(59:3) |
| TAG83 |  | TAG(60:1) |
| TAG84 |  | TAG(60:2) |
| TAG85 |  | TAG(60:3) |
| TAG86 |  | TAG(60:4) |
| TAG87 |  | TAG(62:1) |
| TAG88 |  | TAG(62:2) |
| TAG89 |  | TAG(62:3) |
| TAG90 |  | TAG(62:4) |
| TAG91 |  | TAG(64:2) |
| TAG92 |  | TAG(64:3) |
| TAG93 |  | TAG(40:1) |
| TAG94 |  | TAG(42:3) |
| TAG95 |  | TAG(46:0) |
| TAG96 |  | TAG(46:1) |
| TAG97 |  | TAG(46:3) |
| TAG98 |  | TAG(46:4)A |
| TAG99 |  | TAG(48:2)A |
| TAG100 |  | TAG(48:2)B |
| TAG101 |  | TAG(48:4) |
| TAG102 |  | TAG(50:2)A |
| TAG103 |  | TAG(50:2)B |
| TAG104 |  | TAG(50:3) |
| TAG105 |  | TAG(50:4)A |
| TAG106 |  | TAG(50:4)B |
| TAG107 |  | TAG(54:6)B |
| TAG108 |  | TAG(56:5) |
| TAG109 |  | TAG(56:7) |
| TAG110 |  | TAG(58:4)A |
| TAG111 |  | TAG(58:8) |
| TAG112 |  | TAG(58:9) |
| TAG113 |  | TAG(48:3);TAG(14:1/16:0/18:2) |
| TAG114 |  | TAG(56:2);TAG(18:1/18:1/20:0) |
| TAG115 |  | TAG(56:7);TAG(16:0/18:1/22:6) |
