# Supplementary material for: Analysis of Malassezia Lipidome Disclosed Differences Among the Species and Reveals Presence of Unusual Yeast Lipids
Source: Front Cell Infect Microbiol. 2020 Jul 15;10:338. doi: 10.3389/fcimb.2020.00338 (PMC7374198; doi:10.3389/fcimb.2020.00338)
Supplement: Supplementary Table 3 — FAHFA species detected by UHPLC/MS. *Abbreviations were adapted from: Kolar et al. (2019), Liberati-Cizmek et al. (2019), Balas et al. (2018), Zhu et al. (2018), Hu et al. (2018), and Kuda et al. (2016). [file Table_3.docx]

**Table S3.** FAHFA species detected by UHPLC/MS. *Abbreviations were adapted from: Kolar et al., 2019, Liberati-Čizmek et al., 2019, Balas et al., 2018, Zhu et al., 2018, Hu et al., 2018, Kuda et al., 2106.

| **Compound Identification** | **Compound** | ***Abbreviation** | **Common or chemical name** |
| --- | --- | --- | --- |
| FAHFA1 | FAHFA(18:0);  FAHFA(11:0/7:0) | **UDAHHA** | Undecanoic acid-hydroxy heptanoic acid |
| FAHFA2 | FAHFA(23:1);  FAHFA(18:1/5:0) | **OAHPNA** | Oleic acid-hydroxy pentanoic acid |
| FAHFA3 | FAHFA(24:0);  FAHFA(16:0/8:0) | **PAHHNA** | Palmitic acid-hydroxy hexanoic acid |
| FAHFA4 | FAHFA(24:1);  FAHFA(16:1/8:0) | **POHHNA** | Palmitoleic acid-hydroxy hexanoic acid |
| FAHFA5 | FAHFA(24:1);  FAHFA(18:1/6:0) | **OAHHNA** | Oleic acid-hydroxy hexanoic acid |
| FAHFA6 | FAHFA(25:0);  FAHFA(16:0/9:0) | **PAHNA** | Palmitic acid-hydroxy nonanoic acid |
| FAHFA7 | FAHFA(25:0);  FAHFA(18:0/7:0) | **SAHHNA** | Stearic acid-hydroxy heptanoic acid |
| FAHFA8 | FAHFA(25:1);  FAHFA(18:1/7:0) | **OAHHNA** | Oleic acid-hydroxy heptanoic acid |
| FAHFA9 | FAHFA(25:2);  FAHFA(18:2/7:0) | **LOHHNA** | Linoleic acid-hydroxy heptanoic acid |
| FAHFA10 | FAHFA(26:0);  FAHFA(10:0/16:0) | **DAHPA** | Decanoic acid-hydroxy palmitic acid |
| FAHFA11 | FAHFA(26:0);  FAHFA(16:0/10:0) | **PAHDA** | Palmitic acid-hydroxy decanoin acid |
| FAHFA12 | FAHFA(26:0);  FAHFA(18:0/8:0) | **SAHONA** | Stearic acid-hydroxy octanoic acid |
| FAHFA13 | FAHFA(26:1);  FAHFA(10:0/16:1) | **DAHPONA** | Decanoic acid-hydroxy palmitoleic acid |
| FAHFA14 | FAHFA(26:1);  FAHFA(18:1/8:0) | **OAHONA** | Oleic acid-hydroxy octanoic acid |
| FAHFA15 | FAHFA(26:2);  FAHFA(18:2/8:0) | **LAHONA** | Linoleic acid-hydroxy octanoic acid |
| FAHFA16 | FAHFA(27:0);FAHFA(18:0/9:0) | **SAHNNA** | Stearic acid-hydroxy nonanoic acid |
| FAHFA17 | FAHFA(27:1);  FAHFA(18:1/9:0) | **OAHNNA** | Oleic acid-hydroxy nonanoic acid |
| FAHFA18 | FAHFA(27:2);  FAHFA(18:2/9:0) | **LAHNNA** | Linoleic acid-hydroxy nonanoic acid |
| FAHFA19 | FAHFA(28:0);  FAHFA(12:0/16:0) | **LAHPA** | Lauric acid-hydroxy palmitic acid |
| FAHFA20 | FAHFA(28:0);  FAHFA(18:0/10:0) | **SAHDA** | Stearic acid-hydroxy decanoin acid |
| FAHFA21 | FAHFA(28:1);  FAHFA(10:0/18:1) | **DAHOA** | Decanoic acid-hydroxy oleic acid |
| FAHFA22 | FAHFA(28:1);  FAHFA(12:0/16:1) | **DDAHPOA** | Dodecanoic acid-hydroxy palmitoleic acid |
| FAHFA23 | FAHFA(36:2);  FAHFA(18:1/18:1) | **OAHOA** | Oleic acid-hydroxy oleic acid |
| FAHFA24 | FAHFA(36:3);  FAHFA(18:1/18:2) | **OAHLA** | Oleic acid-hydroxy linoleic acid |
| FAHFA25 | FAHFA(36:4);  FAHFA(18:1/18:3) | **OAHALA** | Oleic acid-hydroxy alpha linolenic acid |
| FAHFA26 | FAHFA(36:4);  FAHFA(18:2/18:2) | **LAHLA** | Linoleic acid-hydroxy linoleic acid |
| FAHFA27 | FAHFA(36:5);  FAHFA(18:3/18:2) | **ALAHLA** | Alpha linolenic acid-hydroxy linoLeic acid |
| FAHFA28 | FAHFA(36:6);  FAHFA(18:3/18:3) | **ALAHALA** | Alpha linolenic acid-hydroxy linoleic acid |
| FAHFA29 | FAHFA(38:2);  FAHFA(18:1/20:1) | **OAHEA** | Oleic acid-hydroxy eicosenoic acid |
| FAHFA30 | FAHFA(38:4);  FAHFA(18:2/20:2) | **LAHEDA** | Linoleic acid-hydroxy eicosadienoic acid |
| FAHFA31 | FAHFA(38:4);  FAHFA(20:3/18:1) | **ETHOA** | Eicosatrienoic acid-hydroxy oleic acid |
| FAHFA32 | FAHFA(38:5);  FAHFA(20:4/18:1) | **AAHOA** | Arachidonic acid-hydroxy oleic acid |
| FAHFA33 | FAHFA(30:3);  FAHFA(14:1/16:2) | **PHHHDA** | Physeteric acid-hydroxy hexadecadienoic acid |
| FAHFA34 | FAHFA(31:0);  FAHFA(15:0/16:0) | **PDAHPA** | Pentadecylic acid-hydroxy palmitic acid |
| FAHFA35 | FAHFA(31:1);  FAHFA(15:0/16:1) | **PDAHPO** | Pentadecylic acid-hydroxy palmitoleic acid |
| FAHFA36 | FAHFA(32:0);  FAHFA(16:0/16:0) | **PAHPA** | Palmitic acid-hydroxy palmitic acid |
| FAHFA37 | FAHFA(32:1);  FAHFA(14:0/18:1) | **MAHOA** | Myristic acid-hydroxy oleic acid |
| FAHFA38 | FAHFA(32:1);  FAHFA(16:1/16:0) | **POHPA** | Palmitoleic acid-hydroxy palmitic acid |
| FAHFA39 | FAHFA(32:2);  FAHFA(14:1/18:1) | **PHHOA** | Physeteric acid-hydroxy oleic acid |
| FAHFA40 | FAHFA(32:2);  FAHFA(16:0/16:2) | **PAHHDA** | Palmitic acid-hydroxy hexadecadienoic acid |
| FAHFA41 | FAHFA(32:2);  FAHFA(16:1/16:1) | **POHPO** | Palmitoleic acid-hydroxy palmitoleic acid |
| FAHFA42 | FAHFA(32:2);  FAHFA(18:1/14:1) | **OAHPHA** | Oleic acid-hydroxy physeteric acid |
| FAHFA43 | FAHFA(32:3);  FAHFA(16:1/16:2) | **POHHDDA** | Palmitoleic acid-hydroxy hexadecadienoic acid |
| FAHFA44 | FAHFA(32:4);  FAHFA(16:3/16:1) | **HDTAHPA** | Hexadecatrienoic acid-hydroxy palmitoleic acid |
| FAHFA45 | FAHFA(32:5);  FAHFA(16:3/16:2) | **HDTAHDDA** | Hexadecatrienoic acid-hydroxy hexadecadienoic acid |
| FAHFA46 | FAHFA(32:5);  FAHFA(16:4/16:1) | **HDTAHPO** | Hexadecatetraenoic acid-hydroxy palmitoleic acid |
| FAHFA47 | FAHFA(33:0);  FAHFA(16:0/17:0) | **PAHMAA** | Palmitic acid-hydroxy margaric acid |
| FAHFA48 | FAHFA(33:1);  FAHFA(17:1/16:0) | **HDAHPA** | Heptadecenoic acid-hydroxy palmitic acid |
| FAHFA49 | FAHFA(33:2);  FAHFA(18:1/15:1) | **OAHPDA** | Oleic acid-hydroxy pentadecenoic acid |
| FAHFA50 | FAHFA(33:3);  FAHFA(17:1/16:2) | **HDAHHDDA** | Heptadecenoic acid-hydroxy hexadecadienoic acid |
| FAHFA51 | FAHFA(34:0);  FAHFA(18:0/16:0) | **SAHPA** | Stearic acid-hydroxy palmitic acid |
| FAHFA52 | FAHFA(34:1);  FAHFA(18:1/16:0) | **OAHPA** | Oleic acid-hydroxy palmitic acid |
| FAHFA53 | FAHFA(34:2);  FAHFA(16:1/18:1) | **POHOA** | Palmitoleic acid-hydroxy oleci acid |
| FAHFA54 | FAHFA(34:2);  FAHFA(18:2/16:0) | **LAHPA** | Linoleic acid-hydroxy palmitic acid |
| FAHFA55 | FAHFA(34:3);  FAHFA(16:1/18:2) | **POHLA** | Palmitoleic acid-hydroxy linoleic acid |
| FAHFA56 | FAHFA(34:3);  FAHFA(18:1/16:2) | **OAHHDDA** | Oleic acid-hydroxy hexadecadienoic acid |
| FAHFA57 | FAHFA(34:3);  FAHFA(18:3/16:0) | **ALAHPA** | Alpha Linolenic acid-hydroxy almitic acid |
| FAHFA58 | FAHFA(34:4);  FAHFA(18:2/16:2) | **LAHHDDA** | linoleic acid-hydroxy hexadecadienoic acid |
| FAHFA59 | FAHFA(34:5);  FAHFA(18:3/16:2) | **ALAHHDDA** | Alpha linolenic acid-hydroxy hexadecadienoic acid |
| FAHFA60 | FAHFA(35:1);  FAHFA(17:0/18:1) | **MAAHOA** | Margaric acid-hydroxy oleic acid |
| FAHFA61 | FAHFA(35:1);  FAHFA(18:1/17:0) | **OAHMAA** | Oleic acid-hydroxy margaric acid |
| FAHFA62 | FAHFA(35:2);  FAHFA(17:1/18:1) | **HDAHOA** | Heptadecenoic acid-hydroxy oleic acid |
| FAHFA63 | FAHFA(35:2);  FAHFA(18:1/17:1) | **OAHHDA** | Oleic acid-hydroxy heptadecenoic acid |
| FAHFA64 | FAHFA(35:3);  FAHFA(18:2/17:1) | **LAHHDA** | Linoleic acid-hydroxy heptadecenoic acid |
| FAHFA65 | FAHFA(36:0);  FAHFA(18:0/18:0) | **SAHSA** | Stearic acid-hydroxy stearic acid |
| FAHFA66 | FAHFA(36:1);  FAHFA(18:1/18:0) | **OAHSA** | Oleic acid-hydroxy stearic acid |
| FAHFA67 | FAHFA(40:4);  FAHFA(16:0/24:4) | **PAHTTA** | Palmitic acid-hydroxy tetracosatetraenoic acid |
| FAHFA68 | FAHFA(40:7);  FAHFA(22:6/18:1) | **DHAHOA** | Docosahexaenoic acid-hydroxy oleic acid |
| FAHFA69 | FAHFA(42:1);  FAHFA(18:1/24:0) | **OAHLGA** | Oleic acid-hydroxy lignoceric acid |
| FAHFA70 | FAHFA(42:5);  FAHFA(18:1/24:4) | **OAHTTA** | Oleic acid-hydroxy tetracosatetraenoic acid |
| FAHFA71 | FAHFA(42:6);  FAHFA(18:2/24:4) | **LAHTTA** | Linoleic acid-hydroxy tetracosatetraenoic acid |
| FAHFA72 | FAHFA(44:1);  FAHFA(26:0/18:1) | **CAHOA** | Cerotic acid-hydroxy oleic acid |
| FAHFA73 | FAHFA(30:0);  FAHFA(14:0/16:0) | **MAHPA** | Myristic acid-hydroxy palmitic acid |
| FAHFA74 | FAHFA(34:0);  FAHFA(16:0/18:0) | **PAHSA** | Palmitic acid-hydroxy stearic acid |
| FAHFA75 | FAHFA(34:1);  FAHFA(16:0/18:1) | **PAHOA** | Palmitic acid-hydroxy oleic acid |
| FAHFA76 | FAHFA(36:3);  FAHFA(18:2/18:1) | **LAHOA** | Linoleic acid-hydroxy oleic acid |
| FAHFA77 | FAHFA(36:4);  FAHFA(20:4/16:0) | **AAHPA** | Arachidonic acid-hydroxy palmitic acid |
