# Supplementary material for: Analysis of Malassezia Lipidome Disclosed Differences Among the Species and Reveals Presence of Unusual Yeast Lipids
Source: Front Cell Infect Microbiol. 2020 Jul 15;10:338. doi: 10.3389/fcimb.2020.00338 (PMC7374198; doi:10.3389/fcimb.2020.00338)
Supplement: Supplementary file 4 [file Data_Sheet_1.docx]

**File 4.** R code. <http://www.R-project.org/>

## -----------------------------------------------------------------------------

# Loading the database

library(here)

lipids <- readRDS(here("data", "ready", "lipids.rds"))

dim(lipids)

## -----------------------------------------------------------------------------

# Picking appropriate colours for species and future plots

mal.col <- c("black", # atypical M. furfur

"darkgoldenrod1", # M. furfur

"yellow4", # globosa

"firebrick", # pachydermatis

"deepskyblue4", # restricta

"navajowhite3" # sympodialis

)

## -----------------------------------------------------------------------------

# Checking the name of the lipids

colnames(lipids)

## -----------------------------------------------------------------------------

# Extracting only those columns with lipids

lpd <- lipids[,3:430] # Metabolome: only the metabolites without strain id

dim(lpd)

## -----------------------------------------------------------------------------

# Turning the Malassezia species into a factor, for future analyses

str <- factor(lipids$strain)

## -----------------------------------------------------------------------------

# Exploratory PCA to check distribution of species in PC space

library(mixOmics)

pca.lpd <- pca(lpd, ncomp = 21, center = TRUE, scale = TRUE)

plot(pca.lpd)

## -----------------------------------------------------------------------------

# Plot exploratory PCA to check distribution of species in PC space

plotIndiv(pca.lpd, group = lipids$strain, ind.names = FALSE,

col.per.group = mal.col,

legend = TRUE, title = 'PCA on the whole lipidome')

## -----------------------------------------------------------------------------

# EXploratory PLS-DA to assess the number of components

lpd.plsda <- plsda(lpd, str, ncomp = 10)

plotIndiv(lpd.plsda , comp = 1:2,

group = str, ind.names = FALSE,

col.per.group = mal.col,

point.lwd = 0.57,

x.label = "PC1",

y.label = "PC2",

ellipse = TRUE, legend = TRUE, title = 'PLS-DA')

## -----------------------------------------------------------------------------

# To check the performance of the initial PLS-DA

set.seed(2543)

# for reproducibility, only when the `cpus' argument is not used

perf.lpd.plsda <- perf(lpd.plsda, validation = "Mfold",

folds = 5,

progressBar = FALSE,

nrepeat = 200)

plot(perf.lpd.plsda, col = color.mixo(5:7), sd = TRUE, legend.position = "horizontal")

## ----message=FALSE, warning=FALSE---------------------------------------------

# Tuning the analysis to check the number of PCs to keep

# grid of possible keepX values that will be tested for each component

list.keepX <- c(1:30, seq(40, 400, 10))

tune.splsda.lpd <- tune.splsda(X = lpd, Y = str,

ncomp = 5,

validation = 'Mfold',

folds = 5,

nrepeat = 200, # repet. per fold

progressBar = TRUE,

dist = 'max.dist',

measure = "BER",

test.keepX = list.keepX,

cpus = 2)

## -----------------------------------------------------------------------------

# Error rate per component for the keepX grid

error <- tune.splsda.lpd$error.rate

ncomp <- tune.splsda.lpd$choice.ncomp$ncomp # optimal number of components based on t-tests

ncomp

## -----------------------------------------------------------------------------

# Optimal number of variables to select

select.keepXselect.keepX <- tune.splsda.lpd$choice.keepX[1:ncomp]

## -----------------------------------------------------------------------------

# Check performance of sPLS-DA with selected PCs and variables

splsda.lpd <- splsda(lpd, str, ncomp = ncomp, keepX = select.keepX)

set.seed(40) # for reproducibility, only when the `cpus' argument is not used

# takes about 1 min to run

perf.lpd <- perf(splsda.lpd, validation = "Mfold", folds = 5,

dist = 'max.dist', nrepeat = 200,

progressBar = FALSE)

## -----------------------------------------------------------------------------

# Plotting whether selected principal components are stable

par(mfrow=c(3,2))

plot(perf.lpd$features$stable[[1]], type = 'h', ylab = 'Stability',

xlab = 'Features', main = 'Comp 1', las =2)

plot(perf.lpd$features$stable[[2]], type = 'h', ylab = 'Stability',

xlab = 'Features', main = 'Comp 2', las =2)

plot(perf.lpd$features$stable[[3]], type = 'h', ylab = 'Stability',

xlab = 'Features', main = 'Comp 3', las =2)

plot(perf.lpd$features$stable[[4]], type = 'h', ylab = 'Stability',

xlab = 'Features', main = 'Comp 4', las =2)

plot(perf.lpd$features$stable[[5]], type = 'h', ylab = 'Stability',

xlab = 'Features', main = 'Comp 5', las =2)

## -----------------------------------------------------------------------------

# Plot of the tuning of the sPLS-DA

plot(tune.splsda.lpd, col = color.jet(5))

## -----------------------------------------------------------------------------

# Checking how lipids are correlated with selected PCs

plotLoadings(splsda.lpd, comp = 1, title = 'Loadings on comp 1',

contrib = 'max', method = 'mean', xlim=c(-1,1))

plotLoadings(splsda.lpd, comp = 2, title = 'Loadings on comp 2',

contrib = 'max', method = 'mean', xlim=c(-1,1))

plotLoadings(splsda.lpd, comp = 3, title = 'Loadings on comp 3',

contrib = 'max', method = 'mean', xlim=c(-1,1))

plotLoadings(splsda.lpd, comp = 4, title = 'Loadings on comp 4',

contrib = 'max', method = 'mean', xlim=c(-1,1))

plotLoadings(splsda.lpd, comp = 5, title = 'Loadings on comp 5',

contrib = 'max', method = 'mean', xlim=c(-1,1))

## -----------------------------------------------------------------------------

# Grid of possible keepX values that will be tested for each component

list.keepX2 <- c(1:60) # Avoiding redundant lipids

tune.splsda.lpd2 <- tune.splsda(X = lpd, Y = str,

ncomp = 5,

validation = 'Mfold',

folds = 5,

nrepeat = 200, # repet. per fold

progressBar = TRUE,

dist = 'max.dist',

measure = "BER",

test.keepX = list.keepX2,

cpus = 2)

## -----------------------------------------------------------------------------

# Error rate per component for the keepX grid

error2 <- tune.splsda.lpd2$error.rate

ncomp2 <- tune.splsda.lpd2$choice.ncomp$ncomp # optimal number of components based on t-tests

ncomp2

## -----------------------------------------------------------------------------

# Optimal number of variables to select

select.keepX2

select.keepX2 <- tune.splsda.lpd2$choice.keepX[1:5]

## -----------------------------------------------------------------------------

# Plot tuning of the definitive sPLS-DA

plot(tune.splsda.lpd2, col = color.jet(5))

## -----------------------------------------------------------------------------

# This one will be the definitive sPLS-DA used for the analysis

splsda.lpd2 <- splsda(lpd, str, ncomp = ncomp2, keepX = select.keepX2)

## -----------------------------------------------------------------------------

# Checking performance of the definitive sPLS-DA

set.seed(40) # for reproducibility, only when the `cpus' argument is not used

# takes about 1 min to run

perf.lpd2 <- perf(splsda.lpd2, validation = "Mfold", folds = 5,

dist = 'max.dist', nrepeat = 200,

progressBar = TRUE)

## -----------------------------------------------------------------------------

# Error rate of the definitive sPLS-DA

perf.lpd2$error.rate

plot(perf.lpd2, col = color.mixo(5))

auroc(splsda.lpd2, roc.comp = 5)

## -----------------------------------------------------------------------------

# Exploring distribution of species in the space created by PCs

pc12 <- plotIndiv(splsda.lpd2, comp = c(1,2),

group = str, ind.names = FALSE,

col.per.group = mal.col,

point.lwd = 0.5,

ylim = c(-4,3),

ellipse = TRUE, style = "ggplot2", legend = TRUE,

title = 'sPLS-DA on Lipidome, comp 1 & 2')

## -----------------------------------------------------------------------------

# Exploring distribution of species in the space created by PCs

pc13 <- plotIndiv(splsda.lpd2, comp = c(1,3),

group = str, ind.names = FALSE,

col.per.group = mal.col,

point.lwd = 0.5,

ylim = c(-3,3),

ellipse = TRUE, style = "ggplot2", legend = TRUE,

title = 'sPLS-DA on Lipidome, comp 1 & 3')

## -----------------------------------------------------------------------------

# Exploring distribution of species in the space created by PCs

pc14 <- plotIndiv(splsda.lpd2, comp = c(1,4),

group = str, ind.names = FALSE,

col.per.group = mal.col,

point.lwd = 0.5,

ylim = c(-6,6),

ellipse = TRUE, style = "ggplot2", legend = TRUE,

title = 'sPLS-DA on Lipidome, comp 1 & 4')

## -----------------------------------------------------------------------------

# Exploring distribution of species in the space created by PCs

pc15 <- plotIndiv(splsda.lpd2, comp = c(1,5),

group = str, ind.names = FALSE,

col.per.group = mal.col,

point.lwd = 0.5,

ylim = c(-3,6),

ellipse = TRUE, style = "ggplot2", legend = TRUE,

title = 'sPLS-DA on Lipidome, comp 1 & 5')

## -----------------------------------------------------------------------------

# Checking the stability of the definitive sPLS-DA

par(mfrow=c(3,2))

plot(perf.lpd2$features$stable[[1]], type = 'h', ylab = 'Stability',

xlab = 'Features', main = 'Comp 1', las =2)

plot(perf.lpd2$features$stable[[2]], type = 'h', ylab = 'Stability',

xlab = 'Features', main = 'Comp 2', las =2)

plot(perf.lpd2$features$stable[[3]], type = 'h', ylab = 'Stability',

xlab = 'Features', main = 'Comp 3', las =2)

plot(perf.lpd2$features$stable[[4]], type = 'h', ylab = 'Stability',

xlab = 'Features', main = 'Comp 4', las =2)

plot(perf.lpd2$features$stable[[5]], type = 'h', ylab = 'Stability',

xlab = 'Features', main = 'Comp 5', las =2)

## -----------------------------------------------------------------------------

# Plot correlation between lipids and the PCs in the definitive sPLS-DA

par(mfrow=c(1,1))

plotLoadings(splsda.lpd2, comp = 1, title = 'Loadings on comp 1',

contrib = 'max', method = 'mean', xlim=c(-1,1))

plotLoadings(splsda.lpd2, comp = 2, title = 'Loadings on comp 2',

contrib = 'max', method = 'mean', xlim=c(-1,1))

plotLoadings(splsda.lpd2, comp = 3, title = 'Loadings on comp 3',

contrib = 'max', method = 'mean', xlim=c(-1,1))

plotLoadings(splsda.lpd2, comp = 4, title = 'Loadings on comp 4',

contrib = 'max', method = 'mean', xlim=c(-1,1))

plotLoadings(splsda.lpd2, comp = 5, title = 'Loadings on comp 5',

contrib = 'max', method = 'mean', xlim=c(-1,1))

## ----fig.height=3, fig.width=3------------------------------------------------

# FIGURE 2A. Heat map correlating lipids and species of Malassezia

splsda.lpd2[["names"]][["sample"]] <- str # Identify rows

cim(splsda.lpd2)

## ----fig.height=4, fig.width=4------------------------------------------------

# FIGURE 2B. Network plot of the lipids that best characterise Malasezzia species

network(splsda.lpd2, comp = 1:5, cutoff = 0.52,

color.edge = color.spectral(8),

color.node = c("white", "grey90"),

lwd.edge = 2)

## -----------------------------------------------------------------------------

# Selecting FAHFA lipids

f.lpd <- lipids[,102:179] # Metabolome: only the metabolites without strain id

dim(f.lpd)

## -----------------------------------------------------------------------------

# Exploratory PCA with FAHFA lipids

library(mixOmics)

pca.f.lpd <- pca(f.lpd, ncomp = 14, center = TRUE, scale = TRUE)

plot(pca.f.lpd)

## -----------------------------------------------------------------------------

# Exploratory PLS-DA with FAHFA lipids

f.lpd.plsda <- plsda(f.lpd, str, ncomp = 10)

plotIndiv(f.lpd.plsda , comp = 1:2,

group = str, ind.names = FALSE,

col.per.group = mal.col,

point.lwd = 0.57,

x.label = "PC1",

y.label = "PC2",

ellipse = TRUE, legend = TRUE, title = 'FAHFA PLS-DA')

## -----------------------------------------------------------------------------

# Checking performance of PLS-DA with FAHFA lipids

set.seed(2543) # for reproducibility, only when the `cpus' argument is not used

perf.f.lpd.plsda <- perf(f.lpd.plsda, validation = "Mfold",

folds = 5,

progressBar = FALSE,

nrepeat = 200)

plot(perf.f.lpd.plsda,

col = color.mixo(5:7),

sd = TRUE,

legend.position = "horizontal")

## -----------------------------------------------------------------------------

# Tuning sPLS-DA to check the number of FAHFA lipids to retain

# grid of possible keepX values that will be tested for each component

list.keepX.f <- c(1:40)

tune.splsda.f.lpd <- tune.splsda(f.lpd, str,

ncomp = 9,

validation = 'Mfold',

folds = 5,

nrepeat = 200, # repet. per fold

progressBar = TRUE,

dist = 'max.dist',

measure = "BER",

test.keepX = list.keepX.f,

cpus = 2)

## -----------------------------------------------------------------------------

# Error rate in exploratory sPLS-DA with FAHFA lipids

f.error <- tune.splsda.f.lpd$error.rate # error rate per component for the keepX grid

## -----------------------------------------------------------------------------

# Selecting optimal number of PCs to retain in sPLS-DA with FAHFA lipids

f.ncomp <- tune.splsda.f.lpd$choice.ncomp$ncomp # optimal number of components based on t-tests

f.ncomp

## -----------------------------------------------------------------------------

# Selecting optimal number of lipids to reatin in sPLS-DA with FAHFA lipids

f.select.keepX <- tune.splsda.f.lpd$choice.keepX[1:f.ncomp] # optimal number of variables to select

f.select.keepX

## -----------------------------------------------------------------------------

# Plotting the tuning of sPLS-DA with FAHFA lipids

plot(tune.splsda.f.lpd, col = color.jet(9))

## -----------------------------------------------------------------------------

# Definitive sPLS-DA with FAHFA lipids

splsda.f.lpd <- splsda(f.lpd, str, ncomp = f.ncomp, keepX = f.select.keepX)

## -----------------------------------------------------------------------------

# Exploring distribution of species in the space created by PCs with FAHFA lipids

pc12 <- plotIndiv(splsda.f.lpd, comp = c(1,2),

group = str, ind.names = FALSE,

col.per.group = mal.col,

point.lwd = 0.57,

x.label = "PC1",

y.label = "PC2",

ellipse = TRUE, style = "ggplot2", legend = TRUE,

title = 'sPLS-DA on FAHFA lipids, comp 1 & 2')

## -----------------------------------------------------------------------------

# Exploring distribution of species in the space created by PCs with FAHFA lipids

pc13 <- plotIndiv(splsda.f.lpd, comp = c(1,3),

group = str, ind.names = FALSE,

col.per.group = mal.col,

point.lwd = 0.57,

x.label = "PC1",

y.label = "PC2",

ellipse = TRUE, style = "ggplot2", legend = TRUE,

title = 'sPLS-DA on FAHFA lipids, comp 1 & 3')

## -----------------------------------------------------------------------------

# Exploring distribution of species in the space created by PCs with FAHFA lipids

pc14 <- plotIndiv(splsda.f.lpd, comp = c(1,4),

group = str, ind.names = FALSE,

col.per.group = mal.col,

point.lwd = 0.57,

x.label = "PC1",

y.label = "PC2",

ellipse = TRUE, style = "ggplot2", legend = TRUE,

title = 'sPLS-DA on FAHFA lipids, comp 1 & 4')

## -----------------------------------------------------------------------------

# Exploring distribution of species in the space created by PCs with FAHFA lipids

pc15 <- plotIndiv(splsda.f.lpd, comp = c(1,5),

group = str, ind.names = FALSE,

col.per.group = mal.col,

point.lwd = 0.57,

x.label = "PC1",

y.label = "PC2",

ellipse = TRUE, style = "ggplot2", legend = TRUE,

title = 'sPLS-DA on FAHFA lipids, comp 1 & 5')

## -----------------------------------------------------------------------------

# Checking performance of definitive sPLS-DA with FAHFA lipids

set.seed(40) # for reproducibility, only when the `cpus' argument is not used

# takes about 1 min to run

perf.f.lpd <- perf(splsda.f.lpd, validation = "Mfold",

folds = 5,

dist = 'max.dist', nrepeat = 200,

progressBar = TRUE)

## -----------------------------------------------------------------------------

# Checking error rate of definitive sPLS-DA with FAHFA lipids

perf.f.lpd$error.rate

plot(perf.f.lpd, col = color.mixo(5))

auroc(splsda.f.lpd, roc.comp = 8)

## -----------------------------------------------------------------------------

# Checking stability of PCs in definitive sPLS-DA with FAHFA lipids

par(mfrow=c(3,2))

plot(perf.f.lpd$features$stable[[1]], type = 'h', ylab = 'Stability',

xlab = 'Features', main = 'Comp 1', las =2)

plot(perf.f.lpd$features$stable[[2]], type = 'h', ylab = 'Stability',

xlab = 'Features', main = 'Comp 2', las =2)

plot(perf.f.lpd$features$stable[[3]], type = 'h', ylab = 'Stability',

xlab = 'Features', main = 'Comp 3', las =2)

plot(perf.f.lpd$features$stable[[4]], type = 'h', ylab = 'Stability',

xlab = 'Features', main = 'Comp 4', las =2)

plot(perf.f.lpd$features$stable[[5]], type = 'h', ylab = 'Stability',

xlab = 'Features', main = 'Comp 5', las =2)

## -----------------------------------------------------------------------------

# Plot correlation between PCs and lipids in definitive sPLS-DA with FAHFA lipids

par(mfrow=c(1,1))

plotLoadings(splsda.f.lpd, comp = 1, title = 'Loadings on comp 1',

contrib = 'max', method = 'mean', xlim=c(-1,1))

plotLoadings(splsda.f.lpd, comp = 2, title = 'Loadings on comp 2',

contrib = 'max', method = 'mean', xlim=c(-1,1))

plotLoadings(splsda.f.lpd, comp = 3, title = 'Loadings on comp 3',

contrib = 'max', method = 'mean', xlim=c(-1,1))

plotLoadings(splsda.f.lpd, comp = 4, title = 'Loadings on comp 4',

contrib = 'max', method = 'mean', xlim=c(-1,1))

plotLoadings(splsda.f.lpd, comp = 5, title = 'Loadings on comp 5',

contrib = 'max', method = 'mean', xlim=c(-1,1))

## ----fig.height=3, fig.width=3------------------------------------------------

# FIGURE 3A. Heatmap of lipids and PCs in definitive sPLS-DA with FAHFA lipids

splsda.f.lpd[["names"]][["sample"]] <- str # Identify rows

cim(splsda.f.lpd)

## ----fig.height=4, fig.width=4------------------------------------------------

# FIGURE 3B. Network plot of lipids that characterise species in definitive sPLS-DA with FAHFA lipids

network(splsda.f.lpd, comp = 1:5, cutoff = 0.51,

color.edge = color.spectral(8),

color.node = c("white", "grey90"),

lwd.edge = 2)

## -----------------------------------------------------------------------------

# Loading count of lipid types per species

library(here)

lipitype <- readRDS(here("data", "ready", "lipitype.rds"))

head(lipitype, 2)

## -----------------------------------------------------------------------------

# Tyding of lipid types per species

library(tidyverse)

lipitype <- lipitype %>%

gather(key = type, value = conc, -strain, -repet) %>%

glimpse()

lipitype$conc <- 100*lipitype$conc

## -----------------------------------------------------------------------------

# Summarising frequency of lipid types per species

lipitype_sd <- plyr::ddply(lipitype, c("type", "strain"),

plyr::summarise,

mean_l = mean(conc),

sd_l = sd(conc))

lipitype_sd <- lipitype_sd %>%

mutate(se = sd_l/sqrt(6), # Six was the number of replicates to calculate each mean

ci = 2 * se) # Only the positive bar

lipitype_sd$type <- as.factor(lipitype_sd$type)

head(lipitype_sd)

## -----------------------------------------------------------------------------

# Reordering lipid types according to their frequency

lipitype_sd$type <- reorder(lipitype_sd$type, lipitype_sd$mean_l)

lipitype_sd$strain <- factor(lipitype_sd$strain,

levels = c("globosa", "sympodialis", "restricta",

"furfur", "pachidermatis", "atypica"))

## -----------------------------------------------------------------------------

# Appropriate labels for lipid types and species of Malassezia

library(forcats)

lipitype_sd2 <- lipitype_sd %>%

filter(type %in% c("TAG", "Cholesterol", "DG", "FA", "PC",

"DGTS", "FAHFA", "PE", "Ceramide", "LCP", "CE"))

lipitype_sd2$type %>%

fct_drop() %>%

levels() # to get rid of the filtered out levels

lipitype_sd2$strain2 <- factor(lipitype_sd2$strain, labels = c(

expression(paste(italic("M."), " ", italic("globosa"))),

expression(paste(italic("M."), " ", italic("sympodialis"))),

expression(paste(italic("M."), " ", italic("restricta"))),

expression(paste(italic("M."), " ", italic("furfur"))),

expression(paste(italic("M."), " ", italic("pachydermatis"))),

expression(paste("atypical ", italic("M. "), italic("furfur")))))

# FIGURE 1. Frequency of lipid type per species

p.lipitype <- ggplot(lipitype_sd2, aes(x = type, y = mean_l, fill = type)) +

geom_errorbar(aes(ymin = mean_l - ci, ymax = mean_l + ci),

width=0.2,

position = position_dodge(0.9)) +

geom_bar(stat = "identity", color = "white",

position = position_dodge(0.9)) +

facet_wrap(vars(strain2), nrow = 2, labeller = label_parsed) +

scale_fill_viridis_d() +

coord_flip() +

labs(x = NULL, y = "Concentration (%mol)") +

theme_minimal() +

theme(panel.grid.major.y = element_line(color = "white"),

panel.grid.major.x = element_line(size = 0.25),

strip.text.x = element_text(size = rel(1.1)),

panel.spacing = unit(1.2, "lines"),

legend.position = "none",

)

p.lipitype

## -----------------------------------------------------------------------------

# saving FIGURE 1. Frequency of lipid type per species

ggsave("1_p.lipitype.pdf", width = 7, height = 4.5,

path = "/Users/aat/Dropbox/Arbeit/dWissenschaft/Malassezia/malassezia_lipidomics/figures/")

## ----fig.height=4, fig.width=3------------------------------------------------

# Exploratory plot of frequency of ALL lipid type per species

library(ggplot2)

str(lipitype_sd)

lipitype_sd$strain2 <- factor(lipitype_sd$strain, labels = c(

expression(paste(italic("M."), " ", italic("globosa"))),

expression(paste(italic("M."), " ", italic("sympodialis"))),

expression(paste(italic("M."), " ", italic("restricta"))),

expression(paste(italic("M."), " ", italic("furfur"))),

expression(paste(italic("M."), " ", italic("pachydermatis"))),

expression(paste("atypical ", italic("M. "), italic("furfur")))))

p.lipitypeAll <- ggplot(lipitype_sd, aes(x = type, y = mean_l, fill = type)) +

geom_errorbar(aes(ymin = mean_l - ci, ymax = mean_l + ci),

width=0.2,

position = position_dodge(0.9)) +

geom_bar(stat = "identity", color = "white",

position = position_dodge(0.9)) +

facet_wrap(vars(strain2), nrow = 2, labeller = label_parsed) +

scale_fill_viridis_d() +

coord_flip() +

labs(x = NULL, y = "Concentration (%mol)") +

theme_minimal() +

theme(panel.grid.major.y = element_line(color = "white"),

panel.grid.major.x = element_line(size = 0.25),

strip.text.x = element_text(size = rel(1.1)),

panel.spacing = unit(1.2, "lines"),

legend.position = "none",

)

p.lipitypeAll

## ----fig.height=4, fig.width=3------------------------------------------------

# Exploratory plot of LOG frequency of ALL lipid type per species

p.lipitypeAll_log <- ggplot(lipitype_sd, aes(x = type, y = mean_l, fill = type)) +

geom_errorbar(aes(ymin = mean_l - ci, ymax = mean_l + ci),

width=0.2,

position = position_dodge(0.9)) +

geom_bar(stat = "identity", color = "white",

position = position_dodge(0.9)) +

facet_wrap(vars(strain2), nrow = 2, labeller = label_parsed) +

scale_fill_viridis_d() +

scale_y_continuous(trans = "log10") +

coord_flip() +

labs(x = NULL, y = "Concentration (%mol)") +

theme_minimal() +

theme(panel.grid.major.y = element_line(color = "white"),

panel.grid.major.x = element_line(size = 0.25),

strip.text.x = element_text(size = rel(1.1)),

panel.spacing = unit(1.2, "lines"),

legend.position = "none",

)

p.lipitypeAll_log
